# A pan‐cancer atlas of cancer hallmark‐associated candidate driver lncRNAs

**DOI:** 10.1002/1878-0261.12381

**Published:** 2018-10-02

**Authors:** Yulan Deng, Shangyi Luo, Xinxin Zhang, Chaoxia Zou, Huating Yuan, Gaoming Liao, Liwen Xu, Chunyu Deng, Yujia Lan, Tingting Zhao, Xu Gao, Yun Xiao, Xia Li

**Affiliations:** ^1^ College of Bioinformatics Science and Technology Harbin Medical University China; ^2^ Department of Biochemistry and Molecular Biology Harbin Medical University China; ^3^ Department of Neurology The First Affiliated Hospital of Harbin Medical University China; ^4^ Key Laboratory of Cardiovascular Medicine Research Harbin Medical University Ministry of Education China

**Keywords:** cancer hallmark, copy number alteration, driver lncRNA, mutual exclusivity, pan‐cancer atlas

## Abstract

Substantial cancer genome sequencing efforts have discovered many important driver genes contributing to tumorigenesis. However, very little is known about the genetic alterations of long non‐coding RNAs (lncRNAs) in cancer. Thus, there is a need for systematic surveys of driver lncRNAs. Through integrative analysis of 5918 tumors across 11 cancer types, we revealed that lncRNAs have undergone dramatic genomic alterations, many of which are mutually exclusive with well‐known cancer genes. Using the hypothesis of functional redundancy of mutual exclusivity, we developed a computational framework to identify driver lncRNAs associated with different cancer hallmarks. Applying it to pan‐cancer data, we identified 378 candidate driver lncRNAs whose genomic features highly resemble the known cancer driver genes (e.g. high conservation and early replication). We further validated the candidate driver lncRNAs involved in ‘Tissue Invasion and Metastasis’ in lung adenocarcinoma and breast cancer, and also highlighted their potential roles in improving clinical outcomes. In summary, we have generated a comprehensive landscape of cancer candidate driver lncRNAs that could act as a starting point for future functional explorations, as well as the identification of biomarkers and lncRNA‐based target therapy.

**Abbreviations**
BRCA: breast invasive carcinoma
CNA: copy number alteration
COAD: colon adenocarcinoma
DFS: disease‐free survival
FDR: false discovery rate
GBM: glioblastoma multiforme
GO: Gene Ontology
HNSC: head and neck squamous cell carcinoma
HR: hazard ratio
LGG: brain lower grade glioma
lncRNA: Long noncoding RNA
LUAD: lung adenocarcinoma
LUSC: lung squamous cell carcinoma
OS: overall survival
OV: ovarian serous cystadenocarcinoma
PCG: protein‐coding gene
PRAD: prostate adenocarcinoma
READ: rectum adenocarcinoma
RPKM: reads per kilobase per million mapped reads
SKCM: skin cutaneous melanoma
STAD: stomach adenocarcinoma
TCGA: The Cancer Genome Atlas

## 1 Introduction

The development of cancer is driven by somatically acquired genetic alterations (Martincorena *et al*., 2015; Yates and Campbell, 2012; Zhang *et al*., 2017b). Many large‐scale genome characterization efforts have uncovered abundant genetic alterations across cancer genomes, with a subset driving tumorigenesis (Kim *et al*., 2013; Zack *et al*., 2013; Zhang *et al*., 2017a). Recently, intensive attention has been drawn to non‐coding genomic regions (Fredriksson *et al*., 2014) and systematic analysis of these loci reveals thousands of non‐coding transcripts (such as long non‐coding RNAs, lncRNAs) (Huarte, 2015; Xu *et al*., 2016). Accumulating studies have suggested that dysfunctional lncRNAs are widely involved in the initiation and progression of cancer (Huarte, 2015; Schmitt and Chang, 2016), such as *PCAT‐1* for cell proliferation (Prensner *et al*., 2011) and *HOTAIR* for tumor metastasis (Huarte, 2015). However, compared to cancer driver genes, cancer genomic characterization of lncRNAs is still largely lacking.

Recent studies have reported that the genetic alteration of some lncRNAs conferred a selective growth advantage to the cancer cells in which it occurs, playing an important role in the initiation and progression of cancer (Schmitt and Chang, 2016; Yan *et al*., 2015). LncRNA *FAL1* was amplified in more than 30% of ovarian cancers, which could promote cell proliferation by recruiting the chromatin repressor protein *BMI‐1* and inhibiting the expression of *CDKN1A* (Hu *et al*., 2014). Frequent amplification in lncRNA *TERC* caused increased telomerase activity and telomere lengthening, and finally resulted in tumor cell immortality (Kim *et al*., 2015; Wang *et al*., 2013). About 10% of melanomas exhibited focal amplifications of lncRNA *SAMMSON*, which regulated vital mitochondrial functions and increased the clonogenic potential of melanoma cells (Leucci *et al*., 2016). These lncRNAs, referred to as driver lncRNAs, are subject to positive selection and can contribute to various malignant phenotypes during the evolutionary process of cancer (Schmitt and Chang, 2016; Yan *et al*., 2015). Therefore, comprehensive identification of genetically altered driver lncRNAs across distinct tumor types is not only urgently needed, but also may promote new diagnostic and therapeutic strategies for cancer (Yan *et al*., 2015).

In sharp contrast to the functional importance of driver lncRNAs, some lncRNAs have a neutral, or perhaps slightly negative, fitness contribution to the cancer cell and act as passengers. Some passenger lncRNAs also show random or hitchhiking somatic mutations, which can confound the analysis of cancer drivers (Garraway and Lander, 2013; Marx, 2014; Pon and Marra, 2015). To address this challenge, several methods have been proposed. ExInAtor employed a parametric statistical test to identify lncRNAs with excess of exonic mutations, accounting for individual mutational signatures (Lanzos *et al*., 2017). OncodriveFML integrated several functional impact scoring metrics to identify driver regions with functional mutation bias (Mularoni *et al*., 2016). Also, ncdDetect combined mutation frequency alongside their functional impact to search for positive selection signs of driver lncRNAs (Juul *et al*., 2017). All of these methods are designed to identify driver lncRNAs with nucleotide substitutions or indels. Recently, Zhou *et al*. (2017) identified driver lncRNAs by integrating somatic copy number alterations, expression profiles, known cancer genes and statistical controls.

In the present study, we comprehensively characterized the genomic alterations of lncRNAs in 5918 cancer patients across 11 tumor types from The Cancer Genome Atlas (TCGA) project. We revealed a large number of genetically altered lncRNAs with significant effects on their gene expression. Interestingly, such genetically altered lncRNAs were found to be mutually exclusive with well‐known cancer driver genes. The observation of mutual exclusivity motivated us to develop a computational method to distinguish the driver lncRNAs from the passengers. Finally, 378 genetically altered lncRNAs that were mutually exclusive with abroad repertoire of known cancer driver genes and exhibited consistent functional effects on cancer hallmarks were identified in 11 cancer types. We further assayed the candidate driver lncRNAs involved in ‘Tissue Invasion and Metastasis’ in lung adenocarcinoma and breast cancer and compiled a systematic catalogue of hallmark‐associated candidate driver lncRNAs publicly available through DriverLncRNA (http://biocc.hrbmu.edu.cn/DriverLncRNA) to facilitate the experimental exploration, biomarker discovery and development of lncRNA‐based clinical trials.

## 2 Materials and methods

Based on functional redundancy hypothesis of mutual exclusivity in pathways, we developed a four‐step method to identify cancer driver lncRNAs that were mutually exclusive with cancer hallmark‐associated genes by integrating copy number and expression profiles in human cancers.

### 2.1 Constructing copy number alteration profile

We obtained copy number (level 3) and mRNA expression data (level 3) for 11 cancer types [lung adenocarcinoma (LUAD), ovarian serous cystadenocarcinoma (OV), head and neck squamous cell carcinoma (HNSC), stomach adenocarcinoma (STAD), colon adenocarcinoma (COAD) and rectum adenocarcinoma (READ) were merged and formed one cancer sample set (CR), prostate adenocarcinoma (PRAD), lung squamous cell carcinoma (LUSC), skin cutaneous melanoma (SKCM), glioblastoma multiforme (GBM), breast invasive carcinoma (BRCA) and brain lower grade glioma (LGG)] from TCGA (https://tcga-data.nci.nih.gov). The lncRNA annotation (V19) was downloaded from GENCODE (Harrow *et al*., 2012) and the gene annotation was downloaded from NCBI (https://www.ncbi.nlm.nih.gov/gene/). The expression data of lncRNAs across the 11 cancer types were collected from TANRIC (Li *et al*., 2015a). The detailed sample information is shown in Table S1.

For copy number alteration (CNA), we re‐implemented the GISTIC algorithm (Mermel *et al*., 2011) by modifying the reference genome file that contains both protein‐coding gene (PCG) and lncRNA annotations. High‐level amplification and homozygous deletion were retained, and the dominant type of CNA for PCGs/lncRNAs was used. We applied the same filtering criteria to PCGs and lncRNAs. First, we selected PCGs/lncRNAs that were altered in at least 2.5% of samples and showed detectable expression [reads per kilobase per million mapped reads (RPKM)] > 0.3 in at least 30% of the samples (Sanchez‐Garcia *et al*., 2014)]. Next, we screened for PCGs/lncRNAs whose copy number significantly affected their expression levels using a one‐tailed Wilcoxon signed rank test with *P *<* *0.05. On average, 231 lncRNAs and 1425 PCGs per cancer type were found to be altered (with amplifications or deletions) (Table S2). Finally, we constructed a binary copy number alteration profile of these PCGs and lncRNAs, where each row is a patient, each column is a gene and value refers to the copy number status of a gene in a certain sample.

### 2.2 Identification of hallmark‐associated Gene Ontology (GO) terms and hallmark‐associated PCGs

We obtained a list of hallmark‐associated GO terms from previous studies (Hnisz *et al*., 2013; Plaisier *et al*., 2012) and these GO‐terms were called curated GO terms in the present study. Meanwhile, to identify more complete hallmark‐associated GO terms, we downloaded the GO from Synapse (syn1741407) and defined these GO‐terms as candidate GO terms. Then, for a given GO term, its average functional association (termed functional similarity score) with curated GO terms from each cancer hallmark was calculated by semantic similarity using function ‘mgoSim’ in r package ‘GOSemSim’. To determine the threshold of functional similarity score, we calculated the functional similarity score for each curated term with the corresponding hallmark (excluding the queried curated term). The minimized functional similarity score (0.2) was used as the threshold for determining new hallmark‐related GO terms (Fig. S1).

Similarly, PCGs derived from these hallmark‐associated GO terms were considered as hallmark‐associated PCGs. To explore whether these PCGs related to a corresponding hallmark, enrichment analysis was performed in five data sources (KEGG, NCI, Reactome, BioCarta and GenMapp) that were downloaded from Synapse (syn1741407) using a hypergeometric test with a false discovery rate (FDR) < 0.05. We found that these hallmark‐associated PCGs tended to be enriched in corresponding hallmark‐associated pathways (Fig. S2). For example, in GenMapp, PCGs associated with the hallmark ‘Reprogramming Energy Metabolism’ were exclusively enriched in ‘Glycogen Metabolism’.

### 2.3 Constructing hallmark‐associated mutually exclusive networks

To identify cancer driver lncRNAs that are mutually exclusive with cancer‐related genes with functional redundancy, we focused on cancer hallmark associated functions. Specifically, for each cancer type, we constructed 10 hallmark‐associated mutually exclusive networks. For a given cancer hallmark, we first extracted hallmark‐associated PCGs and all lncRNAs from binary copy number alteration profile, then built a 2 × 2 contingency table and investigated their mutually exclusive relationships (including PCG‐PCG, lncRNA‐lncRNA, PCG‐lncRNA). A pair of genes was considered to be mutually exclusive if (i) they were significantly mutually exclusive by means of the hypergeometric test (*P *<* *0.05) or (ii) their co‐occurrence was never observed in any samples (Fig. S3). Finally, the inter‐connected PCGs/lncRNAs were used to build a mutually exclusive network associated with the specific cancer hallmark.

### 2.4 Identifying mutually exclusive modules in a hallmark‐associated context

For each cancer hallmark, we identified all maximal cliques from the corresponding mutually exclusive network using function ‘maximal.cliques’ in r package ‘igraph’. These cliques were regarded as the mutually exclusive seed sets. We started with the seed set and then iteratively expanded the set by a greedy search procedure in order to increase patient coverage. In each iteration, a candidate gene list was generated based on the following criteria: (i) the candidate gene should appear in the binary alteration matrix; (ii) for PCG, it should be associated with the same hallmark as this clique; (iii) the alteration of candidate gene should be significantly mutually exclusive with the alteration of current gene sets, using a hypergeometric test with *P *<* *0.05; (iv) the numbers of the samples with the genetic alteration of both the candidate gene and any current gene should be fewer than those with their unique alterations. Subsequently, a mutually exclusive metric *F* was calculated for candidate genes using Eqn (1)

(1) $$F=\frac{\Gamma \left(M\right)}{\sum \Gamma \left(g_{i}\right)-\Gamma \left(M\right)+\alpha }$$

where *M* represents a seed set, *g*_*i*_ indicates the *i*th gene in the set *M*, Γ(*g*_*i*_) denotes the number of patients in which *g*_*i*_ was altered, and Γ(*M*) denotes the number of patients who harbor genomic alterations in at least one of the genes in *M*. The parameter α was set to 1 to ensure that the denominator would not be zero. We iteratively performed greedy search to maximize *F* for each time until no candidate gene was found. Finally, permutations were carried out to test whether the expended modules were still significantly mutual exclusive. Specifically, we adopted the random strategy of Markov chain Monte Carlo, which preserved patient‐ and gene‐wise alteration rates, and thus considered the overall heterogeneity across samples. We permuted for 1000 times using R package BiRewire (Gobbi *et al*., 2014). Each time, we recalculated *F*. The empirical *P* value was obtained by calculating the fraction of times for which *F* was larger than the real one. In addition, extended modules with Bonferroni correction FDR < 0.05 were considered as candidate mutually exclusive modules.

### 2.5 Functional assessment of mutually exclusive modules

To further confirm functional associations of the mutually exclusive modules with cancer hallmarks, we assessed the functional effects of the mutually exclusive modules. For each mutually exclusive module, we divided samples into two groups: one with genomic alterations in at least one of the members in the module and the other without. The differentially expressed PCGs between the two groups were then identified using the Bioconductor packages edger (Robinson *et al*., 2010) and limma (Ritchie *et al*., 2015) with voom (Law *et al*., 2014) correction (FDR < 0.05). The functions enriched by these differentially expressed PCGs (hypergeometric test with FDR < 0.05) were determined. If the enriched functions (at least one of the enriched GO terms) were exactly associated with the corresponding hallmark, the mutually exclusive module was kept as a hallmark‐associated mutually exclusive module. Also, lncRNAs in those modules were considered to play important roles in corresponding cancer hallmarks.

### 2.6 Exclusion of potential confounders

To exclude potential confounders induced by proximal known cancer drivers, we extracted 235 candidate driver lncRNAs with any transcripts overlapping protein‐coding genes on the opposite strand, or within 10 kb at their closest point on the same strand (Lanzos *et al*., 2017). In addition, their overlapped (or proximal) PCGs were also extracted. Subsequently, we investigated whether these overlapped (or proximal) PCGs were known drivers in corresponding cancer types. In detail, for each cancer type, only copy number affected PCGs that were recorded in the known driver PCG category downloaded from Cancer Gene Census (CGC) (Futreal *et al*., 2004) were regarded as driver PCGs according to the following criteria: (i) the copy number alteration of drivers occurred in more than 2.5% of samples; (ii) the copy number alteration showed detectable RNA expression (RPKM > 0.3 in at least 30% of the samples); and (iii) the copy number alteration significantly affected RNA expression levels by one‐tailed Wilcoxon signed rank test with *P *<* *0.05. If the overlapped (or proximal) PCGs are known drivers in corresponding cancer type, the lncRNAs identified may act as passengers. Finally, we removed some of these potential false positives that have been reported to promote tumorigenesis.

### 2.7 Cancer associated PCGs and disease associated lncRNAs

Cancer associated PCGs were obtained from Cancer Gene Census (Futreal *et al*., 2004). To determine whether PCGs from mutually exclusive modules were significantly overlapped with cancer associated PCGs, we first random selected the same number of PCGs from candidate PCGs (i.e. PCGs appeared in binary alteration matrix and were hallmark‐associated) 1000 times. Then, empirical *P* value was obtained by calculating the fraction of times in which overlap was higher than that in the real data. Disease associated lncRNAs were obtained from Lnc2Cancer (Ning *et al*., 2016). We combined driver lncRNAs identified from all of the 11 cancers, and the significance of overlap between driver lncRNAs and disease‐associated lncRNAs was evaluated by a hypergeometric test.

### 2.8 Average size of clusters in protein–protein interaction networks

Protein–protein interaction networks were obtained from STRING, version 9 (Search Tool for the Retrieval of Interacting Genes/Proteins) (Franceschini *et al*., 2013). For each cancer, we mapped PCGs from mutually exclusive modules onto the protein–protein interaction network and calculated the cluster size. To determine whether PCGs from mutually exclusive modules showed close functional similarity, we random selected the same number of PCGs from candidate PCGs (i.e., PCGs appeared in binary alteration matrix and were hallmark‐associated) 1000 times, and the empirical *P* value was obtained by calculating the fraction of times in which average size of clusters was larger than that in the real data (Andrews *et al*., 2015).

### 2.9 Properties of driver lncRNAs

The evolutionary conservation was evaluated by 46‐way phastCons vertebrate conserved elements from UCSC (Siepel *et al*., 2005). We computed average phastCons scores for exon regions of lncRNA. The comparison of evolutionary conservation between lncRNAs from modules and the other lncRNAs was performed by a two‐tailed Wilcoxon signed rank test. Similar tests were carried out for cancer associated PCGs.

Sensitive/ultra‐sensitive regions were extracted from a previous study (PMID: 24092746) and the lncRNAs for which its exon regions overlapped with those regions were identified. Then, enrichment for lncRNAs from modules in these lncRNAs was evaluated by a hypergeometric test. Similar tests were carried out for cancer associated PCGs.

Replication timings were acquired from a previous study (Lawrence *et al*., 2013). The comparison of replication timings of lncRNAs from modules and other lncRNAs was evaluated by a two‐tailed Wilcoxon signed rank test. Similar tests were carried out for cancer associated PCGs. As for replication timing data from UCSC ENCODE tracks for MCF‐7, SK‐N‐SH, HepG2, IMR90, HUVEC, NHEK, K562 and Hela‐S3 cell lines, we calculated the early‐to‐late (E/L) ratio as (G1b+S1)/(S4 + G2) averaged over the gene and lncRNA length as in a previous study (Li *et al*., 2015b). Early and late replicated genes denote genes or lncRNAs with an E/L ratio > 1 or < 1 for all eight cell lines, respectively. For aggregation analysis, we considered a lncRNA/gene as an early‐replicated lncRNA/gene if it was early‐replicated in all eight cell lines, with a similar consideration for a late‐replicated lncRNA/gene. Enrichment for driver lncRNAs/cancer associated PCGs in early replicated lncRNAs/genes was evaluated by a hypergeometric test.

Genome‐wide DNase I‐hypersensitive sites (DHSs) of 125 cell lines were directly extracted from UCSC genome browser (http://hgdownload.cse.ucsc.edu/goldenPath/hg19/encodeDCC/wgEncodeRegDnaseClustered/). We extracted lncRNAs that overlapped with DHS in all 125 cell lines, and enrichment for overlap was evaluated by a hypergeometric test. Similar tests were carried out for cancer associated PCGs.

### 2.10 Tissue‐specificity analysis

Raw RNA‐seq data in 16 normal tissues were obtained from Illumina Human Body Map Project (HBM) (https://www.illumina.com). The SRA file for each tissue was downloaded and converted to FASTQ format using the sra toolkit (https://www.ncbi.nlm.nih.gov/sra/docs/toolkitsoft). Reads were mapped to the human reference genome hg19 using TopHat, version 2.0.13 (https://ccb.jhu.edu/software/tophat) with default parameters (Trapnell *et al*., 2009). The expression of lncRNAs was calculated using the RPKM measure. For each driver lncRNA, we calculated normalization score (Yanai *et al*., 2005) using Eqn (2):

(2) $$\text{normalization score}=\frac{\sum_{i=1}^{n}\left(1-\frac{\exp_{i}}{\exp_{\max}}\right)}{n-1}$$

where *n* was the number of tissues and exp_*i*_ was the expression of lncRNA in *i*th tissue and exp_max_ was the maximal expression of lncRNA among different tissues. The comparison of tissue specificity between driver lncRNAs from multiple cancers and driver lncRNAs from a single cancer was performed by a two‐tailed Wilcoxon signed rank test. To explore the tissue‐specificity of cancer‐specific driver lncRNAs, we focused on whether these cancer‐specific lncRNAs had the ability to distinguish cancer samples of different tissues of origin. Then, a pan‐cancer expression profile of 314 cancer‐specific driver lncRNAs was constructed and transformed by log_10_ (RPKM + 1) (Faino *et al*., 2016; Sun *et al*., 2015). Next, the transformed expression matrix was used as input to an unsupervised hierarchical clustering algorithm using a Euclidean distance metric and complete linkage. For clustering analysis, heatmaps were produced using the heatmap.2 function from the ggplot package in r.

### 2.11 Analysis of associations with clinical factors

The pathology reports and clinical data, including follow‐up information of 11 major cancer types from TCGA, were downloaded via the cBioPortal website (Cerami *et al*., 2012; Gao *et al*., 2013) (http://www.cbioportal.org) using the r package ‘CGDS’. Patients were stratified based on continuous copy number alteration or expression above or below the median. The survival curves were estimated using the Kaplan–Meier method, and a log rank test was used to analyze differences in survival time. A Cox proportional hazard regression model was used in univariate and multivariate analyses to determine the impact of risk factors on overall survival and disease‐free survival with adjustment for other potential confounding factors: age, gender, grade and histological types.

Additional glioma clinical validation data were downloaded on 1 July 2016 from cBioPortal website (http://www.cbioportal.org), which includes 35 disease‐free patients and 11 disease‐progressed patients who had not been used in primary analysis. Pairwise continuous mean‐segment copy number variation data were downloaded from FireBrowse website (http://firebrowse.org). When a lncRNA overlapped multiple segments, the average of the mean‐segment value was taken. Patients were stratified based on median continuous copy number alteration, and survival curves were estimated using the Kaplan–Meier method.

### 2.12 Identification of meta‐genes

Because genomic proximity invariably and strongly influences the frequency of concurrent events, we thus combined genes that are mutated in nearly the same patients into larger ‘meta‐genes’. Specifically, we first constructed a mutually exclusive network as described in the text. Then, genes located in same chromosome band and shared the same neighborhood in the mutually exclusive network were merged into a ‘meta‐gene’ (Leiserson *et al*., 2013) and a chromosome band combined with a random‐selected PCG or lncRNA that was located in a corresponding band was regarded as a representative gene. The modules size was calculated in a ‘meta‐gene’ manner throughout the text.

### 2.13 Candidate lncRNAs for functional validation

We chose LUAD and BRCA as examples and evaluated oncogenic lncRNA associated with the hallmark ‘Tissue Invasion and Metastasis’ in A549 and MCF‐7, respectively. Candidate lncRNAs were selected as follows: (i) lncRNAs were amplified in LUAD or BRCA and associated with the hallmark ‘Tissue Invasion and Metastasis’. (ii) lncRNAs were amplified in corresponding cells at log_2_ ratio > 0 (i.e., A549 for LUAD and MCF‐7 for BRCA). Here, copy number alterations of lncRNAs in cell lines were obtained from the Cancer Cell Line Encyclopedia (Barretina *et al*., 2012) (https://portals.broadinstitute.org/ccle/home). (iii) Expression of lncRNAs in cell lines was detectable in corresponding cell lines. The expression of lncRNAs in A549 was re‐annotated from GSE15805, as in a previous study (PMID: 23728290). The expression of lncRNAs in MCF‐7 was obtained from ENCODE (Consortium, 2012) (https://www.encodeproject.org/experiments/ENCSR667JTA). (iv) The RefSeq statuses of lncRNAs were validated (https://www.ncbi.nlm.nih.gov/gene).

### 2.14 siRNA transfection

MCF7 and A549 were cultured in DMEM (Gibco, Gaithersburg, MD, USA) supplemented with 10% fetal bovine serum (ScienCell, Carlsbad, CA, USA). All cells were incubated at 37 °C in a humidified incubator containing 5% CO_2_. All siRNA oligonucleotides were signed and provided by GenePharma (Shanghai, China). Transfections were performed using Lipofectamine 3000 (Invitrogen, Carlsbad, CA, USA). Then, 7.5 μL of Lipofectamine 3000 and 75 pmol of siRNA were used for one well of a six‐well plate. The transfected cells were analyzed after 48 h. The siRNA sequences were:

Negative control siRNA:

Sense: 5′‐UUCUCCGAACGUGUCACGUTT‐3′
Antisense: 5′‐ACGUGACACGUUCGGAGAATT‐3′

RP11‐295G20.2 siRNA:

1#Sense: 5′‐GGAACAGUAAAUGGAGUAATT‐3′
Antisense: 5′‐UUACUCCAUUUACUGUUCCTT‐3′
2#Sense: 5′‐UCCAGUCAACCCAAAGAAUTT‐3′
Antisense: 5′‐AUUCUUUGGGUUGACUGGATT‐3′

RP11‐429J17.7 siRNA:

1#Sense: 5′‐CCCAGAUAUCCUCAUCCAUTT‐3′
Antisense: 5′‐AUGGAUGAGGAUAUCUGGGTT‐3′
2#Sense: 5′‐GGAUGAACCAGGCUAUGUUTT‐3′
Antisense: 5′‐AACAUAGCCUGGUUCAUCCTT‐3′

RHPN1‐AS1 siRNA:

1#Sense: 5′‐GGCCGAUGCUUCCAAGUUTT‐3′
Antisense: 5′‐AACUUUGGAAGCAUCGGCCTT‐3′
2#Sense: 5′‐UCUAAAUCCUGAAGGCUAATT‐3′
Antisense: 5′‐UUAGCCUUCAGGAUUUAGATT‐3′

RP11‐98D18.9:

1#Sense: 5′‐GCAUAUGGCGAUGAGGACUTT‐3′
Antisense: 5′‐AGUCCUCAUCGCCAUAUGCTT‐3′
2#Sense: 5′‐ACCAGCAGCAGAUCAAGAATT‐3′
Antisense: 5′‐UUCUUGAUCUGCUGCUGCUGGUTT‐3′

### 2.15 RNA isolation and real‐time quantitative PCR

Total RNA was extracted using TRIzol reagent (Invitrogen). cDNA was generated using EasyScript One‐Step gDNA Removal and cDNA Synthesis SuperMix Kit (TransGene Biotech, Mallampet, Hyderabad, India) in accordance with the manufacturer's instructions. Real‐time RT‐PCR was performed using SYBR Green reagents (Applied Biosystems, Foster City, CA, USA) in accordance with manufacturer's instructions. Changes in lncRNA levels were determined by the 2^−ΔΔCT^ method using GAPDH as the internal control. The primers used for qRT‐PCR were:

RP11‐295G20.2: Forward primer: 5′‐ACAAGGCATGTTCTGCTCTG‐3′

Reverse primer: 5′‐AAATTGAAAGTGGGAAGACCA‐3′

RP11‐429J17.7: Forward primer: 5′‐TCTCCCTATAATCATTCACAAG‐3′

Reverse primer: 5′‐CCAGCAAATCCCTCCTCT‐3′

RHPN1‐AS1: Forward primer: 5′‐GCTCCTGGTCATCAAGTTCCTCT‐3′

Reverse primer: 5′‐GCACAGGCACCAGAATGATCC‐3′

RP11‐98D18.9: Forward primer: 5′‐GGGCGATGCTCCATCAGTT‐3′

Reverse primer: 5′‐GCCTCCGCAAAGGAATAGAA‐3′

GAPDH: Forward primer: 5′‐CGCTCTCTGCTCCTCCTGTT‐3′

Reverse primer: 5′‐CCATGGTGTCTGAGCGATGT‐3′

### 2.16 Transwell migration assay

The Transwell migration assay was performed using a Transwell system (Corning Inc., Lowell, MA, USA). About 600 μL of complete medium was added to the lower chamber in a 24‐well plate, and 200 μL of cell suspension (3 × 10^4^ cell) in serum‐free medium was added to the upper chamber. After incubating for 24 h (37 °C, 5% CO_2_), the membrane was taken out and fixed with 4% paraformaldehyde for 10 min at room temperature. After fixation, cells were stained with 0.5% crystal violet for 15 min.

## 3 Results

### 3.1 LncRNAs are extensively impacted by copy number alterations

**Figure 1:**
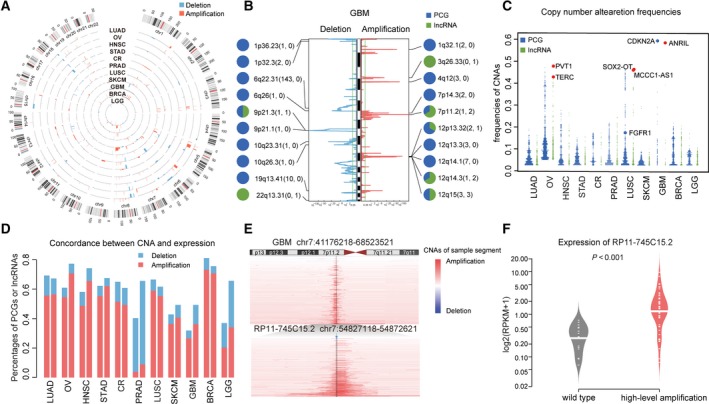
Copy number alterations of lncRNA in cancers. (A) A genome‐wide view of CNAs in lncRNA‐containing loci in cancers. Each track shows the frequency of lncRNA CNAs in one cancer type. (B) The lncRNAs and PCGs in the top 10 representative peaks in GBM. The numbers of PCGs (left) and lncRNAs (right) in each peak are indicated in parentheses. Pie chart of each peak shows the proportion of PCG and lncRNA in the peak. (C) The frequencies of CNAs for lncRNAs and PCGs across cancers. (D) Percentages of PCGs (left) and lncRNAs (right) with concordant CNA and mRNA expression. (E) Copy number profiles of chromosome 7p (upper) and a zoom‐in region (lower) from GBM specimens. The positions of lncRNA RP11‐745C15.2 are noted with black vertical lines. (F) RP11‐745C15.2 expression levels in amplified and normal samples in GBM.

Copy number alterations affected a large fraction of cancer genomes, activating oncogenes and inactivating tumor suppressors, and consequently contributed to tumorigenesis. To characterize the CNAs of lncRNAs, we analyzed single nucleotide polymorphism arrays of 5918 tumor samples across 11 cancer types from TCGA project. As a result, 414 recurrently amplified and 290 recurrently deleted regions, referring to a total 10 392 lncRNAs, were identified across the 11 cancer types (Fig. 1A). An average of 3080 lncRNAs (and 4038 PCGs) per cancer type showed copy number alterations. Especially, there were 74 lncRNAs located in 47 recurrent regions without any PCGs, such as chromosome 3q26.33 and 22q13.31 in GBM (Figs 1B and S4). By calculating the CNA frequencies of lncRNAs for each cancer type, we observed that the alteration frequencies of lncRNAs were comparable to those of PCGs across cancers (Fig. 1C). Remarkably, some lncRNAs exhibited extremely high frequencies of CNAs, even exceeding those of many known driver PCGs. For example, the lncRNA with the highest alteration frequency in LUSC, *SOX2‐OT*, was amplified in 45.6% of samples, which is higher than the cancer gene *FGFR1* with frequency of 17.8%. Also, the intergenic lncRNA *PVT1* showed the highest genomic amplification frequency in OV, covering 47.2% of samples. Other frequently altered lncRNAs included *ANRIL* (deleted in 58.5% samples of GBM), *MCCC1‐AS1* (amplified in 46.1% samples of LUSC) and *TERC* (amplified in 41.6% samples of OV).

Furthermore, we estimated the contribution of CNAs to lncRNA dysregulation by analyzing the correlation between lncRNA copy number and RNA expression level in each cancer type. About 60% of those lncRNAs exhibited positive correlations between their expression and their copy numbers across cancers, comparable to an average of 64.8% for PCGs (Fig. 1D), implying that copy number alteration was a potent contributor to lncRNA dysregulation in cancer. For example, lncRNA *RP11‐745C15.2* was amplified in 39.1% of samples in GBM, resulting in an almost 50‐fold increase in the expression compared to wild‐type (Fig. 1E,F). Overall, our results showed that, similar to PCGs, lncRNAs were extensively affected by CNAs, suggesting their potential oncogenic roles in tumorigenesis and cancer progression.

### 3.2 Systematically identifying mutually exclusive modules in cancer

**Figure 2:**
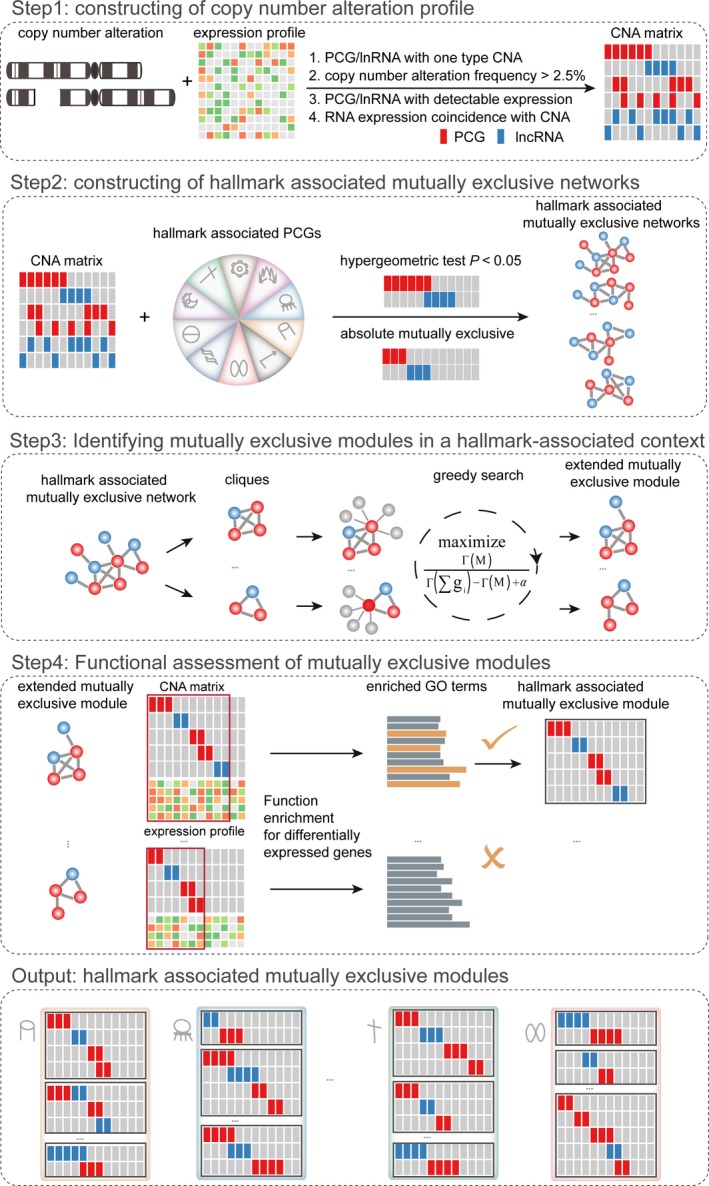
The overview of identification of hallmark‐associated mutually exclusive modules.

Mutual exclusivity among PCGs with frequent CNAs has been widely reported (Babur *et al*., 2015; Ciriello *et al*., 2012; Zhao *et al*., 2012). They often form mutually exclusive modules that deregulate common downstream biological pathways in human cancers, leading to the hypothesis of functional redundancy of mutual exclusivity (Ciriello *et al*., 2012; Sparks *et al*., 1998). We thus speculated that such patterns could be extrapolated to non‐coding RNAs, which will help to clarify the oncogenic roles of lncRNAs. To answer the question, we proposed a four‐step method to identify mutually exclusive modules among lncRNAs and PCGs. In brief, a genomic alteration profile including copy number changes of PCGs and lncRNAs was first constructed by integrating genomic and transcriptomic data. Then, by combining cancer hallmarks and the genomic alteration profile, we constructed 10 cancer hallmark‐associated mutually exclusive networks. For each hallmark‐associated mutually exclusive network, we identified significantly mutually exclusive modules using a greedy search algorithm, and further assessed their affected functions. Finally, mutually exclusive modules that were associated with various cancer hallmarks were established (Fig. 2).

Applying the method to the 11 cancer types, we identified about 200 cancer hallmark‐associated mutually exclusive modules per cancer type (the sizes of modules ranged from 2 to 6) (Fig. S5A), involving a total of 1109 PCGs and 385 lncRNAs (Table S3). Notably, 60.5% of the modules contained lncRNAs, of which 19.4% included more than two lncRNAs. To exclude potential confounders induced by known cancer drivers, we carefully checked lncRNAs that were located close to (or partially overlapped with) them, leaving 378 lncRNAs. Furthermore, in each cancer, we separately permuted CNA profile and expression profile by directly permuting sample labels for PCGs or lncRNAs. For each type of randomization, we performed the corresponding randomization procedure 100 times. Then, we re‐identified the predicted number of lncRNA candidate drivers using our method. We found that the number of lncRNA candidate drivers discovered here, in each cancer type, was significantly greater than random chance (*P *<* *0.05, permutation test) (Fig. S6). The *P* values that associated mutually exclusive modules with cancer hallmark closely followed the expected uniform distribution (Fig. S7), suggesting that low false positive prediction rates.

**Figure 3:**
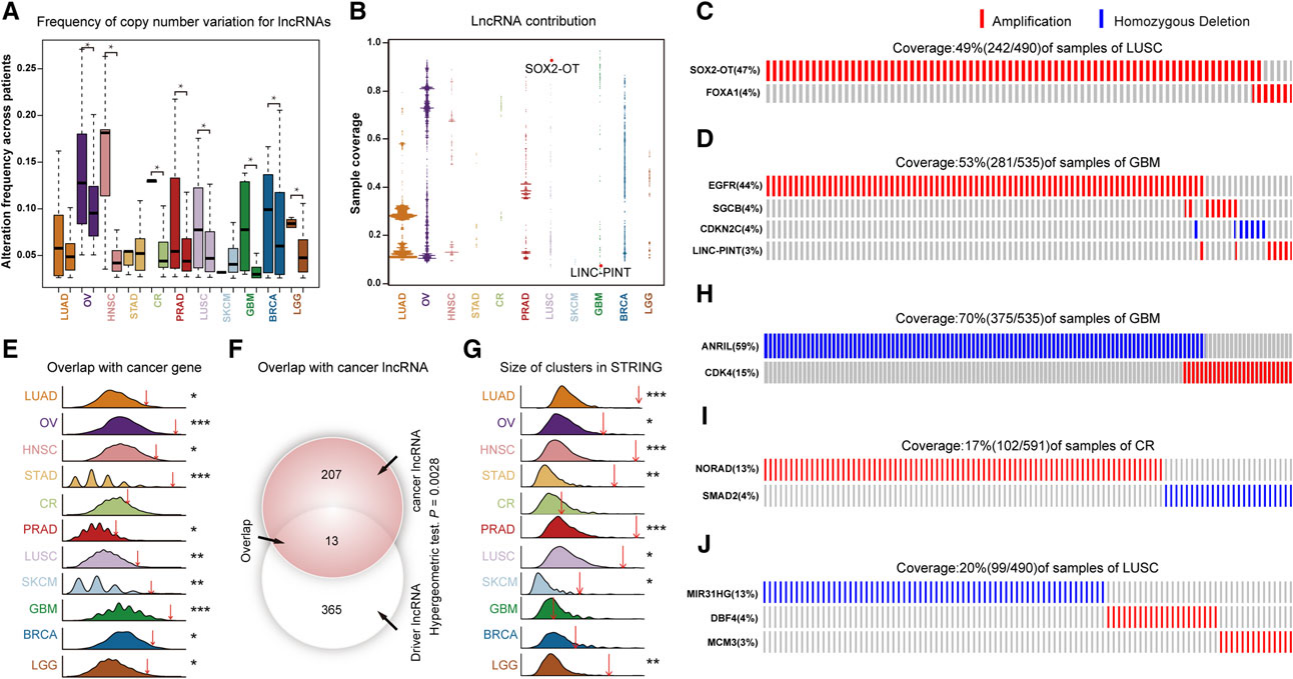
The PCGs and lncRNAs in mutually exclusive modules across 11 major cancer types. (A) The frequencies of CNA for lncRNAs from modules (left) and other lncRNAs (right) (*P <* *0.05, permutation test). (B) Percentages of sample covered by lncRNAs in the modules. (C,D) The modules of lncRNA *SOX2‐OT* and *LINC‐PINT*. Each column represents three tumor samples. (E) The percentage of PCGs from modules (red arrow) and from permutations (curve) that overlap with cancer‐associated PCGs (*P <* *0.05, **P <* *0.01, ***P <* *0.001, permutation test). (F) The overlap between lncRNAs from modules (*n* = 378) and cancer‐associated lncRNAs (*n* = 220) with 13 869 lncRNAs as background (hypergeometric test). (G) The size of clusters in STRING for PCGs from modules (red arrow) and permutations (curve) (*P <* *0.05, **P <* *0.01, ***P <* *0.001, permutation test). (H–J) The modules of lncRNA ANRIL, NORAD and MIR31HG from GBM, CR and LUSC, respectively. Each column represents three tumor samples for GBM and one tumor sample for CR and LUSC.

Similar to PCGs, in most of cancer types, these lncRNAs tended to harbor significant higher alteration frequencies than other lncRNAs (Figs 3A and S5B). Moreover, lncRNAs from the mutually exclusive modules accounted for, on average, more than 51.8% of samples. We further used the fraction of samples explained by lncRNAs in modules to characterize the contribution of lncRNAs in each mutual module. We found the contributions of lncRNAs varied dramatically across 11 cancer types (Fig. 3B). For example, amplification of lncRNA *SOX2‐OT* was mutually exclusive with *FOXA1* with contribution reaching 92.1% in LUSC (Fig. 3C), whereas amplification of lncRNA *LINC‐PINT* was mutually exclusive with *EGFR*,* SGCB* and *CDKN2C* in GBM with only 5.4% of contribution (Fig. 3D). Notably, both of the two lncRNAs have been reported to be associated with cancer. The overexpression of *SOX2‐OT* increased the colony formation ability, promoted cell cycle and facilitated mobility in lung adenocarcinoma cell line (Saghaeian Jazi *et al*., 2016). *LINC‐PINT* was confirmed to promote cell proliferation and survival by regulating the expression of genes of the *TGF‐b*,* MAPK* and p53 pathways (Marin‐Bejar *et al*., 2013). Taken together, these observations indicated that lncRNAs like PCGs were also involved in forming mutually exclusive modules.

### 3.3 Genetically altered lncRNAs are frequently mutually exclusive with known cancer driver genes

Importantly, we observed that PCGs in these cancer hallmark‐associated mutually exclusive modules were significantly enriched for known cancer driver genes in most cancer types (*P *=* *0.027, *P *<* *0.001, *P *=* *0.012, *P *=* *0.01, *P *=* *0.007, *P *=* *0.022, *P *<* *0.001, *P *=* *0.049, *P *<* *0.001 and *P *=* *0.002 for BRCA, GBM, HNSC, LGG, LUSC, LUAD, OV, PRAD, SKCM and STAD, respectively, permutation test) (Fig. 3E). The same results were also observed by significance enrichment analyses (*P *=* *0.01, *P *<* *0.001, *P *=* *0.003, *P *=* *0.01, *P *<* *0.001, *P *=* *0.01, *P *=* *0.02, *P *=* *0.02, *P *=* *0.008 and *P *=* *0.003 for GBM, LUSC, HNSC, BRCA, OV, LUAD, CR, SKCM, LGG and STAD, respectively, hypergeometric test) (Fig. S8). Likewise, the lncRNAs in the modules also showed a significant enrichment for disease‐associated lncRNAs derived from Lnc2Cancer (Ning *et al*., 2016) (*P *<* *0.003, hypergeometric test) (Fig. 3F). Furthermore, we compared our results with candidate driver lncRNAs predicted by previous studies (Iyer *et al*., 2015; Liu *et al*., 2017; Zhu *et al*., 2016). Three previous studies identify cancer specific lncRNAs or functional lncRNAs through differentially expressed transcript analysis (‘MiTranscriptome’), or CRISPR screening strategy [Liu *et al*. (CRISPRi) and Zhu *et al*. (CRISPRd)]. We found that 71 lncRNAs were identified in both our results and MiTranscriptome (*P *=* *6.28 × 10^–15^, hypergeometric test) (Table S4). Besides, the candidates were also significantly enriched in MiTranscriptome cancer specific lncRNAs for five cancer types (*P *=* *0.037, *P *<* *0.001, *P *=* *0.009, *P *=* *0.002, *P *=* *0.032, for HNSC, LUSC, LUAD, PRAD and STAD, respectively, hypergeometric test) (Table S4). For CRISPRi, we totally extracted 286 functional lncRNAs, among which 27 lncRNAs were identified by our study (*P *=* *1.97 × 10^–8^, hypergeometric test) (Table S5). When comparing results using cancer type‐matched cell lines (i.e. U87 for GBM, MCF7 and MDA‐MB‐231 for BRCA), we observed statistically significant enrichments for BRCA (*P *=* *0.005, hypergeometric test) (Table S5) but not for GBM. Although CRISPRd identified functional lncRNAs in the liver cancer cell line Huh7.5OC, we only found two candidates identified in our results (amplification of lncRNA *LINC00885* and *AC084809.2* in HNSC and BRCA, respectively; *P *=* *0.30, hypergeometric test), which may be a result of the tissue specificity of lncRNAs. Also, our results were significantly enriched in lncRNAs located in focal genomic alteration peaks identified by Yan *et al*. (2015) (*P *=* *4.24 × 10^–4^, hypergeometric test). In conjunction with the literature, we further confirmed that many lncRNAs were mutually exclusive with many well‐known cancer driver PCGs. For example, deletion of lncRNA *ANRIL* and *CDK4* constituted a mutually exclusive module associated with the hallmark ‘Self Sufficiency in Growth Signals’ in GBM (Fig. 3H). *CDK4* was amplified frequently in several cancers and was considered to be essential for the initiation of cell cycle (Hamilton and Infante, 2016). In line with this, *ANRIL* was reported to promote cell proliferation (Huarte, 2015). LncRNA *NORAD*, amplified in 13% of samples in colorectal cancer, was mutually exclusive with *SMAD2* (Fig. 3I). *SMAD2* is a member of mitotic checkpoints, and contributes to chromosomal instability in nasopharyngeal carcinoma (Wang *et al*., 2000). The inactivation of *NORAD* was sufficient to produce a chromosomal instability phenotype (Lee *et al*., 2016). In LUSC, deletion of lncRNA *MIR31HG* was identified to form a mutually exclusive module affecting hallmark ‘Genome Instability and Mutation’ (Fig. 3J). In the mutually exclusive module, one member PCG *DBF4* plays an important role in the initiation of DNA replication (Abbas *et al*., 2013) and another member PCG *MCM3* can induce replication stress (Alvarez *et al*., 2015). Consistently, the lncRNA *MIR31HG* was also found to repress a DNA replication stress‐associated protein *INK4A* (Montes *et al*., 2015). In addition, by mapping the PCGs in the modules onto the protein–protein interaction networks, we found that, in most cancer types, they were highly connected to each other (Fig. 3G), supporting their functional associations. Taken together, these findings showed that genetically altered lncRNAs were highly mutually exclusive with well‐known cancer driver genes, suggesting the high associations of these mutually exclusive modules with cancer and highlighting functional redundancy of mutual exclusivity.

### 3.4 Cancer driver gene‐like genomic properties of lncRNAs from mutually exclusive modules

**Figure 4:**
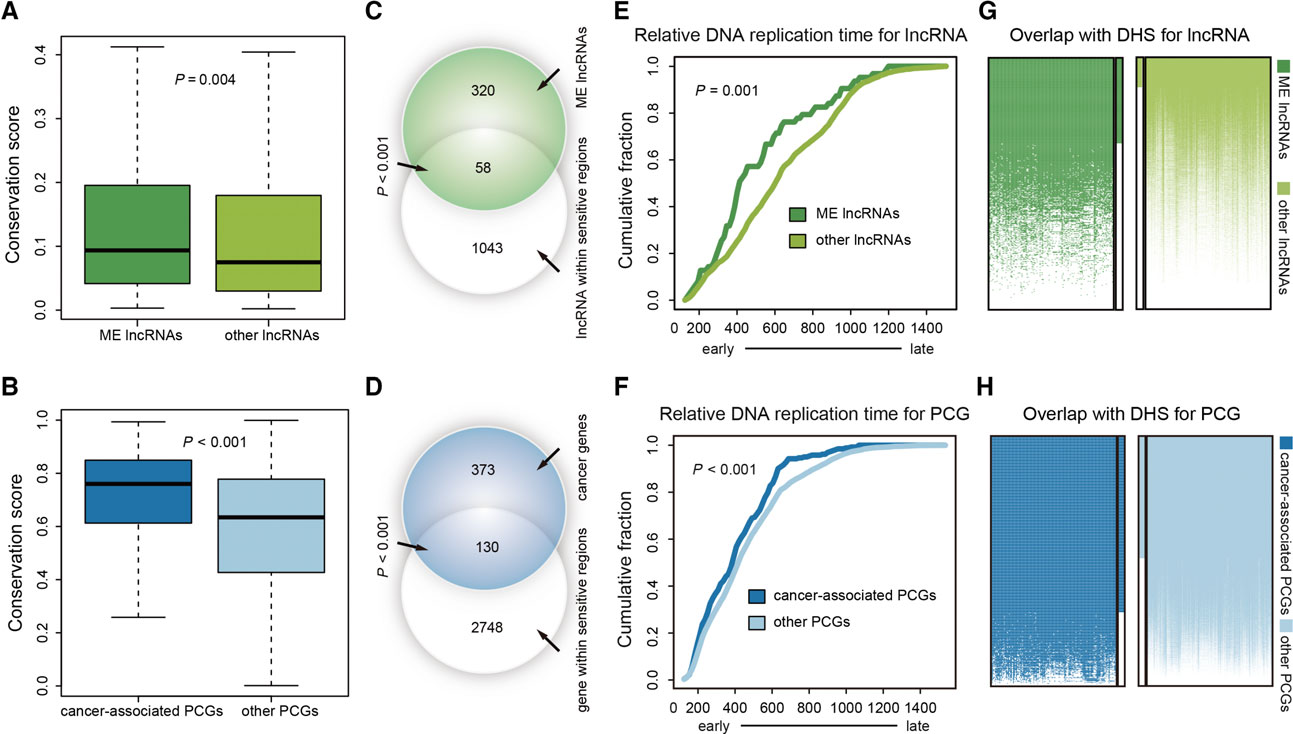
Properties of driver lncRNAs: conservation, chromatin accessibility and early replication. (A,B) Comparison of phastCons score between lncRNAs from modules/cancer‐associated PCGs and other lncRNAs/PCGs. (C,D) Venn diagrams showing the overlap of lncRNAs (*n* = 1101)/PCGs (*n* = 503) located in sensitive regions and lncRNAs from modules (*n* = 378)/cancer‐associated PCGs (*n* = 2878) with 13 869 lncRNAs or 18 992 PCGs as background. (E,F) Cumulative distribution of replication timing is shown for lncRNAs from modules/cancer‐associated PCGs and other lncRNAs/PCGs. (G,H) Heatmaps show the overlap between lncRNAs from modules/cancer‐associated PCGs and DHS from 125 cell lines. Each row indicates a lncRNA/PCG, and each column indicates a cell line. The vertical bars beside heatmaps indicate the percentage of lncRNAs/PCGs that overlaps with DHS in all of the 125 cell lines. The ME lncRNAs in figures indicate lncRNAs from mutually exclusive modules.

We further investigated whether lncRNAs from the cancer hallmark‐associated mutually exclusive modules shared important genomic properties with well‐known cancer driver PCGs. We found that lncRNAs from the mutually exclusive modules showed significantly higher genomic conservation than the other lncRNAs, resembling the known cancer driver PCGs (*P *=* *0.004 and *P *<* *0.001, respectively, two‐tailed Wilcoxon signed rank test) (Fig. 4A,B). Similarly, these lncRNAs and cancer driver PCGs were both significantly enriched in sensitive/ultra‐sensitive regions, which exhibit depletion of common polymorphisms and strong enrichment in disease‐causing mutations (Khurana *et al*., 2013) (*P *<* *0.001, hypergeometric test) (Fig. 4C,D), suggesting that these lncRNAs from the mutually exclusive modules were under strong purifying selection.

Accumulating evidence shows that replication timing shapes the landscape of tumorigenesis (Woo and Li, 2012) and many known cancer driver genes are enriched in early‐replicating regions (Woo and Li, 2012). We thus explored replication timing of the lncRNAs from the mutually exclusive modules using build‐in replication timing data of MutSigCV (Lawrence *et al*., 2013). Consistent with cancer driver PCGs, these lncRNAs were frequently located in early replicating regions (*P *=* *0.001 and *P *<* *0.001, respectively, two‐tailed Wilcoxon signed rank test) (Fig. 4E,F). The same results were also observed by analyzing the Repli‐seq data in tissue matching cell lines from UCSC (*P *<* *0.001, hypergeometric test) (Fig. S9). In addition, using ENCODE epigenetic data, we observed that these lncRNAs, similar to known cancer driver PCGs, showed a high enrichment of DNase I hypersensitive sites (*P *<* *0.001, hypergeometric test) (Fig. 4G,H), which has been suggested to be aberrantly regulated during carcinogenesis (Jin *et al*., 2015).

Taken together, similar to known cancer driver PCGs, lncRNAs from the cancer hallmark‐associated mutually exclusive modules were under strong purifying selection, intolerant to common polymorphisms, replicated earlier, and displayed more open chromatin accessibility, strongly implying their driver roles in tumorigenesis.

### 3.5 Candidate driver lncRNAs contribute to hallmark functions in a cancer‐specific manner

**Figure 5:**
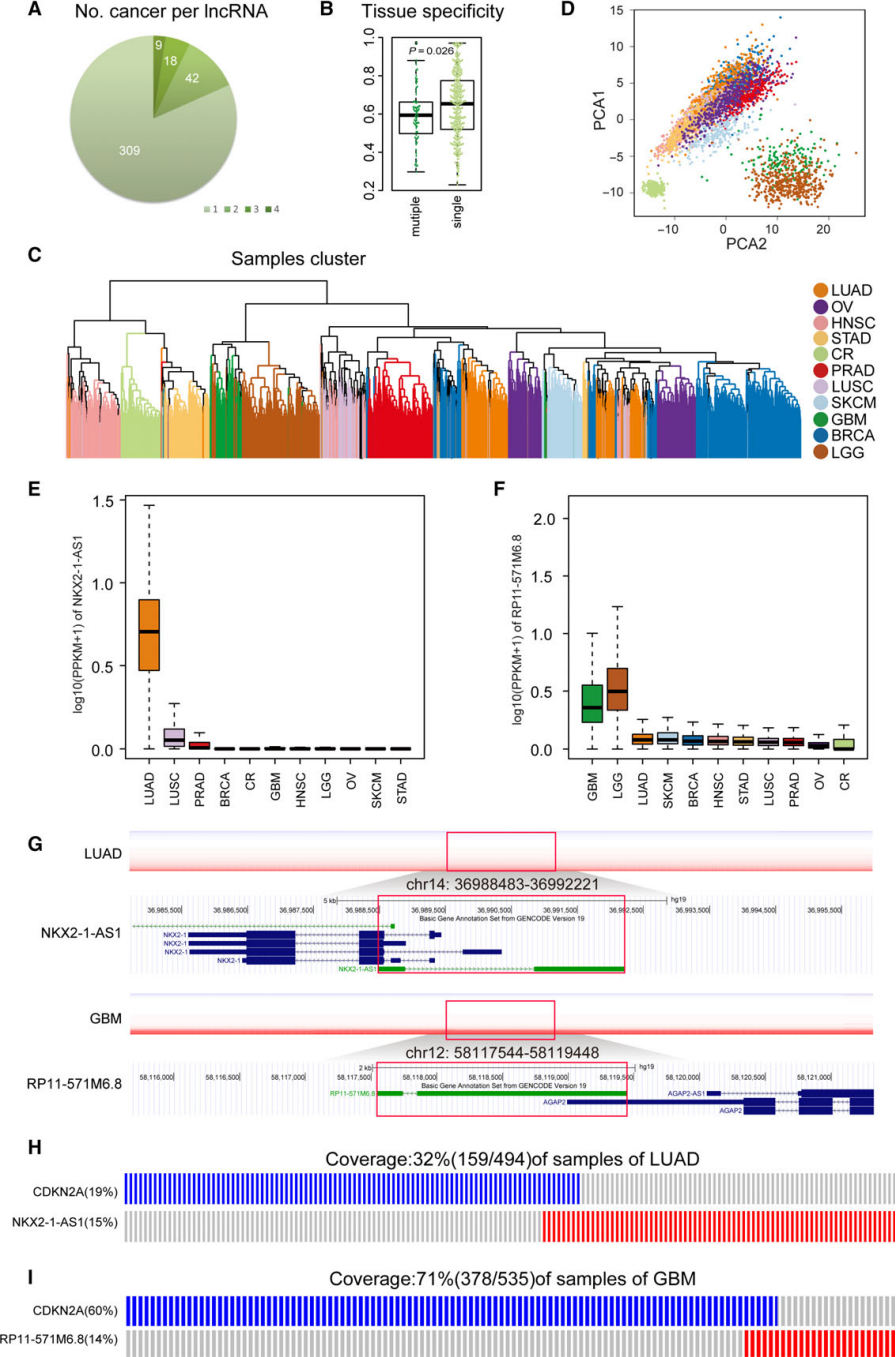
Cancer‐specific driver lncRNAs. (A) The distribution of driver lncRNAs that were identified from single or multiple cancers. (B) The difference of tissue specificity score between cancer‐specific driver lncRNAs and common driver lncRNAs. (C) Hierarchical clustering of the expression data based on cancer‐specific driver lncRNA. (D) Principal component analysis of cancer‐specific driver lncRNAs showing clustering of tumors from the same or related cancer types. (E,F) Box plots showing the expression of cancer‐specific lncRNA *NKX2‐1‐AS1* (LUAD) and *RP11‐571M6.8* (GBM). (G) Copy‐number profiles of NKX2‐1‐AS1 (upper) and RP11‐571M6.8 (lower), respectively, from LUAD and GBM specimens and their genome browser shots. The positions of lncRNA NKX2‐1‐AS1 and RP11‐571M6.8 are shown inside the red boxes. (H,I) The mutually exclusive modules of cancer‐specific lncRNA *NKX2‐1‐AS1* (upper) and lncRNA RP11‐571M6.8 (lower). Each column represents three tumor samples for GBM and one for LUAD.

We next analyzed the distribution of candidate driver lncRNAs across various cancer types. As a result, up to 81.7% (309/378) of candidate driver lncRNAs occurred in only one cancer type, whereas 18.3% (69/378) were identified in multiple cancer types (Fig. 5A). Tissue specificity analysis indicated that cancer‐specific candidate driver lncRNAs showed significantly higher tissue‐specific expression levels compared to common candidate lncRNAs (*P *=* *0.029, two‐tailed Wilcoxon signed rank test) (Fig. 5B), indicating a tissue‐specific pattern of cancer‐specific candidate driver lncRNAs. An unsupervised hierarchical clustering based on the expression of these cancer‐specific candidate driver lncRNAs successfully clustered cancer patients according to tissue type (Fig. 5C). Principle component analysis further confirmed the highly significant correlations among cancers with different tissues of origin (Fig. 5D).

For example, a LUAD specific lncRNA *NKX2‐1‐AS1* showed a 4.53‐fold increase of expression in LUAD compared to other cancer types (*P* < 0.001, two‐tailed Wilcoxon signed rank test) (Fig. 5E). A GBM specific lncRNA *RP11‐571M6.8* displayed a more than 2.7‐fold increase of expression in neural tumors relative to other cancer types (*P *<* *0.001, two‐tailed Wilcoxon signed rank test) (Fig. 5F). Interestingly, these two candidate driver lncRNAs *RP11‐571M6.8* and *NKX2‐1‐AS1* were mutually exclusive with a common gene *CDKN2A* with frequent genetic alterations (> 10%) (Fig. 5G–I) in GBM and LUAD, respectively, affecting the same cancer hallmark ‘Insensitivity to Antigrowth Signals’. Indeed, more than 60.8% (188/309) of specific candidate driver lncRNAs had common mutually exclusive partners, affecting the same hallmarks, in different cancer types, highlighting the functional importance of tissue specific lncRNAs. Moreover, these cancer specific lncRNAs could also participate in unique hallmark functions that are essential in corresponding cancer types. For example, in GBM, amplification of *RP11‐745C15.2* and deletion of *CDKN2B‐AS1* were both associated with the hallmark pathway of ‘ceramide biosynthetic process’, which was reported to promote apoptosis in glioblastoma (Sordillo *et al*., 2016), whereas, in PRAD, deletion of lncRNA *AC003102.3*,* RGMB‐AS1*, and *DLG5‐AS1* was all related to the hallmark pathway of ‘Urogenital System Development’, during which the dysfunction in cell lineage specification predisposed prostate epithelia to hyperplasia and cancer (Brechka *et al*., 2016). These findings suggested that these specific lncRNAs play important roles in contributing to the deregulation of tissue‐specific functions during the tumorigenesis.

**Figure 6:**
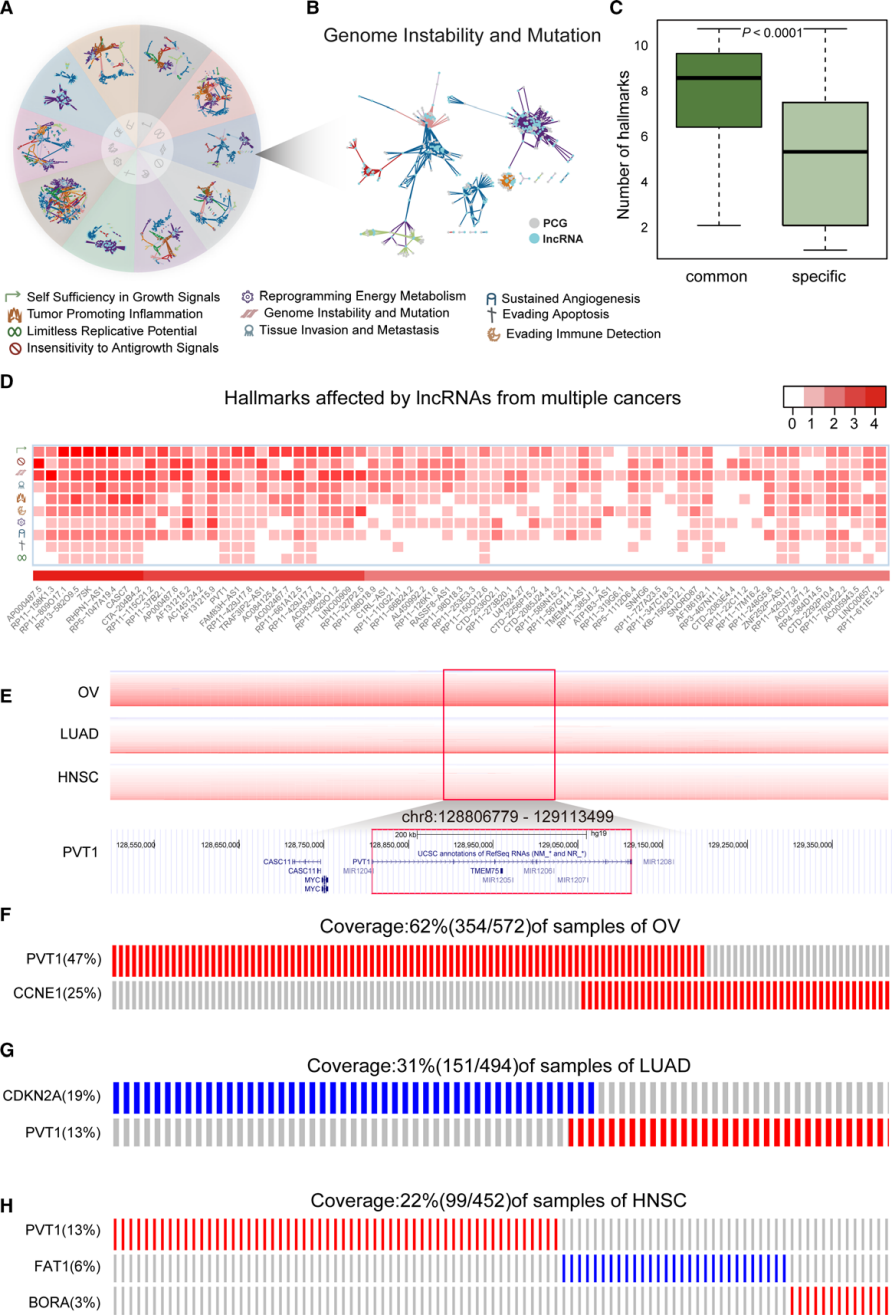
Common driver lncRNAs. (A,B) Mutually exclusive relationship from modules associated with each cancer hallmark. (C) Box plots showing the difference of the number of hallmarks affected by cancer‐specific driver lncRNAs and common driver lncRNAs. (D) Hallmarks affected by common driver lncRNAs. Intensity of the red color corresponds to the number of cancers shown in the legend. (E) Copy number profiles of PVT1 from OV, LUAD and HNSC specimens and its genome browser shot. The positions of lncRNA PVT1 are shown inside the red boxes. (F–H) The mutually exclusive modules of lncRNA PVT1 from LUAD, HNSC and OV, respectively. Each column represents three tumor samples for OV: two for LUAD and one for HNSC.

In comparison to specific lncRNAs, the common candidate driver lncRNAs affected more hallmark functions (*P *<* *0.001, two‐tailed Wilcoxon signed rank test) (Figs 6C,D and S10). A common candidate driver lncRNA *PVT1* was found to be associated with all of the 10 hallmarks (Fig. S11), among which ‘Genome Instability and Mutation’ (Tseng *et al*., 2014), ‘Self Sufficiency in Growth Signals’ (Cui *et al*., 2016), ‘Evading Apoptosis’ (Liu *et al*., 2015) and ‘Tissue Invasion and Metastasis’ (Liu *et al*., 2015) have been validated previously. Moreover, 92.8% (64/69) of these common candidate lncRNAs affect the same hallmarks in multiple cancers (Fig. 6D). To our surprise, no common mutually exclusive partners were observed for any common driver lncRNA when affecting the same hallmark (Fig. 6A,B). For example, the common candidate driver lncRNA *PVT1* was associated with the hallmark ‘Genome Instability and Mutation’ in three cancer types (OV, LUAD and HNSC). In OV, *PVT1* was mutually exclusive with *CCNE1* (Fig. 6E,F) and deregulation of *CCNE1* expression led to genomic instability via mitotic delay (Caldon *et al*., 2013). However, in LUAD, *PVT1* exhibited mutually exclusive with *CDKN2A* (Fig. 6E,G), which induced DNA replication stress and subsequently contributed to genomic instability. In HNSC, *PVT1*,* BORA* and *FAT1*, constituted a mutually exclusive module (Fig. 6E,H), among which, *BORA* was involved in repair of double‐strand breaks (Cairns *et al*., 2015). This suggested that common candidate driver lncRNAs formed mutually exclusive modules with different PCGs in different cancers to affect the same cancer hallmarks.

### 3.6 Clinical benefits of candidate driver lncRNAs

**Figure 7:**
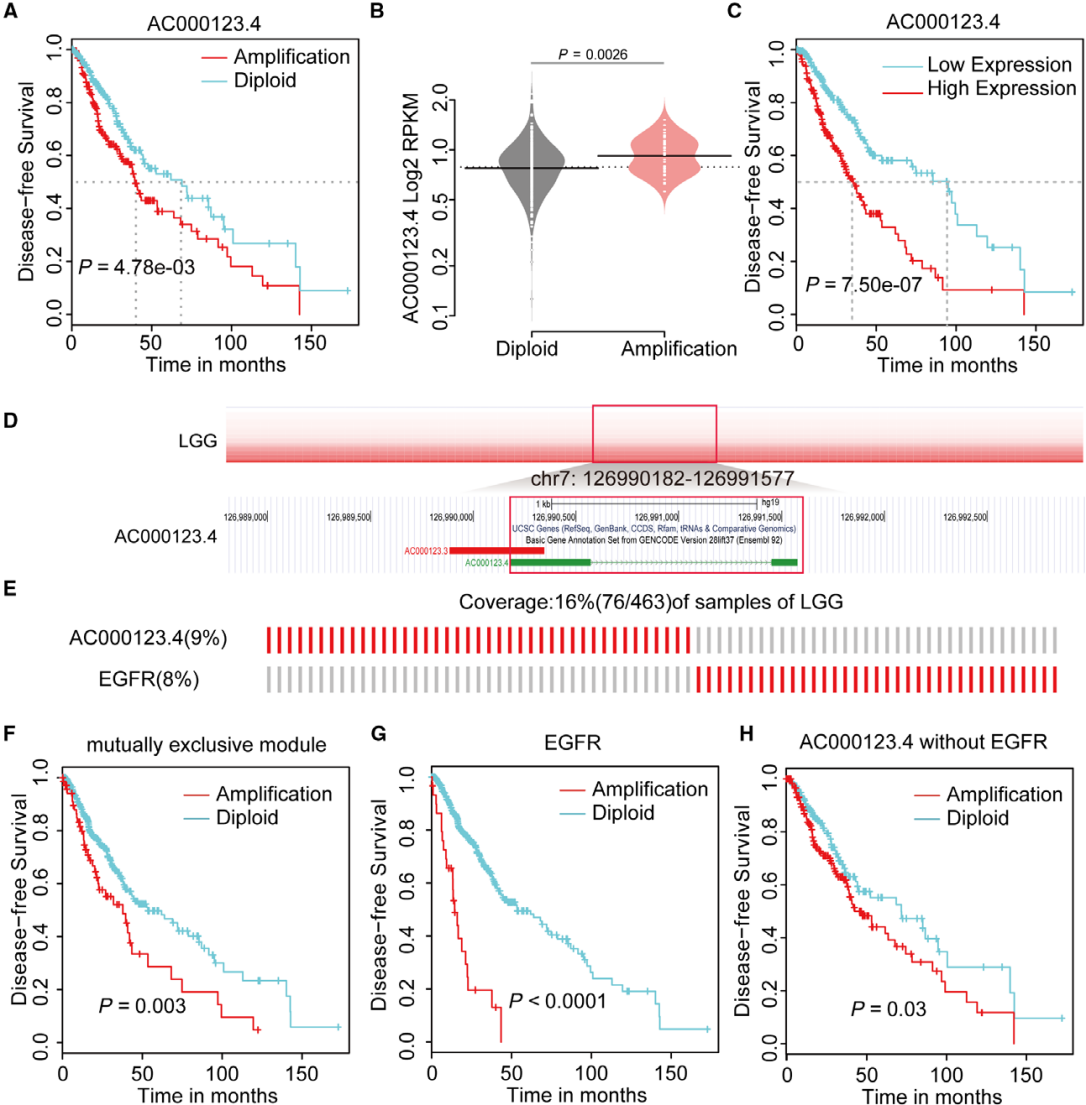
Clinical benefits of driver lncRNAs. (A) Kaplan–Meier plot of DFS of LGG patients grouped by copy number status of lncRNA *AC000123.4*. (B) The expression of AC000123.4 was correlated with copy number alterations. (C) Kaplan–Meier plots of DFS of LGG patients grouped by expression of lncRNA *AC000123.4*. (D) Copy number profile of AC000123.4 from LGG specimens and its genome browser shot. The positions of lncRNA AC000123.4 are shown inside the red boxes. (E) The mutually exclusive module of lncRNA *AC0001*23.4 and *EGFR*. (F) The samples covered by mutually exclusive modules containing *AC000123.4* and *EGFR*, showing poor prognosis. (G) Kaplan–Meier plots of DFS of LGG patients grouped by copy number status of *EGFR*. (H) Kaplan–Meier plots of DFS of LGG patients without *EGFR* alteration, grouped by copy number status of *AC000123.4*.

To assess whether candidate driver lncRNAs can be used to improve clinical outcome, we performed univariate and multivariate Cox regression analyses to evaluate their independent prognostic significance for overall survival (OS) and disease‐free survival (DFS). We identified 71 candidate driver lncRNAs that were significantly predictive of OS or DFS (Table S6). Specifically, in LGG, amplification of candidate driver lncRNA *AC000123.4* conferred a poor prognosis to patients with glioma (*P *<* *0.001 for DFS, log‐rank test) (Fig. 7A). The median recurrence‐free interval of *AC000123.4* amplification patients was 39.6 months [95% confidence interval (CI) = 35.6–63.8], whereas that of *AC000123.4* diploid patients was 68.9 months (95% CI = 44.5–100.9). Multivariate Cox proportional hazards models further showed that *AC000123.4* amplification had a poor effect on DFS (hazard ratio (HR), 1.99, 95% CI = 1.38–2.88) (Table S7) independent of the patient's age, gender and pathologic stages. Moreover, its expression was significantly up‐regulated in tumors with *AC000123.4* amplification (*P *=* *0.002, Student's t test) (Fig. 7B). Likewise, we observed that up‐expression of *AC000123.4* in LGG was also significantly associated with decreased survival in patients (*P *<* *0.001 for DFS, log‐rank test) (Fig. 7C), independent of the patient's age, gender and pathologic stages (Table S8). Additional data also supported the prognostic relevance of *AC000123.4* amplification for DFS (Fig. S12).

In our results, the amplification of *AC000123.4* was mutually exclusive with *EGFR* (Fig. 7D,E), affecting hallmark ‘Sustained Angiogenesis’. Their constituted mutually exclusive module was associated with poor survival (Fig. 7F). Activated *EGFR* increased the production of tumor‐derived *VEGF* that acts on endothelial cells in a paracrine manner to promote angiogenesis (Larsen *et al*., 2011) and amplification of *EGFR* also indicated poor prognosis in glioma (Sun *et al*., 2014) (Fig. 7G). Interestingly, in *EGFR* wild‐type samples, amplification of *AC000123.4* is associated with a worse prognosis (DFS: HR = 1.86, 95% CI = 1.26–2.76, *P *=* *0.0018) (Fig. 7H and Table S9), independent of the patient's age, gender and pathologic stages. The median recurrence‐free interval of *AC000123.4* amplification patients without *EGFR* amplification was 42.9 months (95% CI = 38.90–74.8), whereas that of *AC000123.4* diploid patients without *EGFR* amplification was 72.0 months (95% CI = 44.55–not reached), suggesting a complementary prognostic role of *AC000123.4* to *EGFR*.

### 3.7 DriverLncRNA: a comprehensive landscape of hallmark‐associated candidate driver lncRNAs

**Figure 8:**
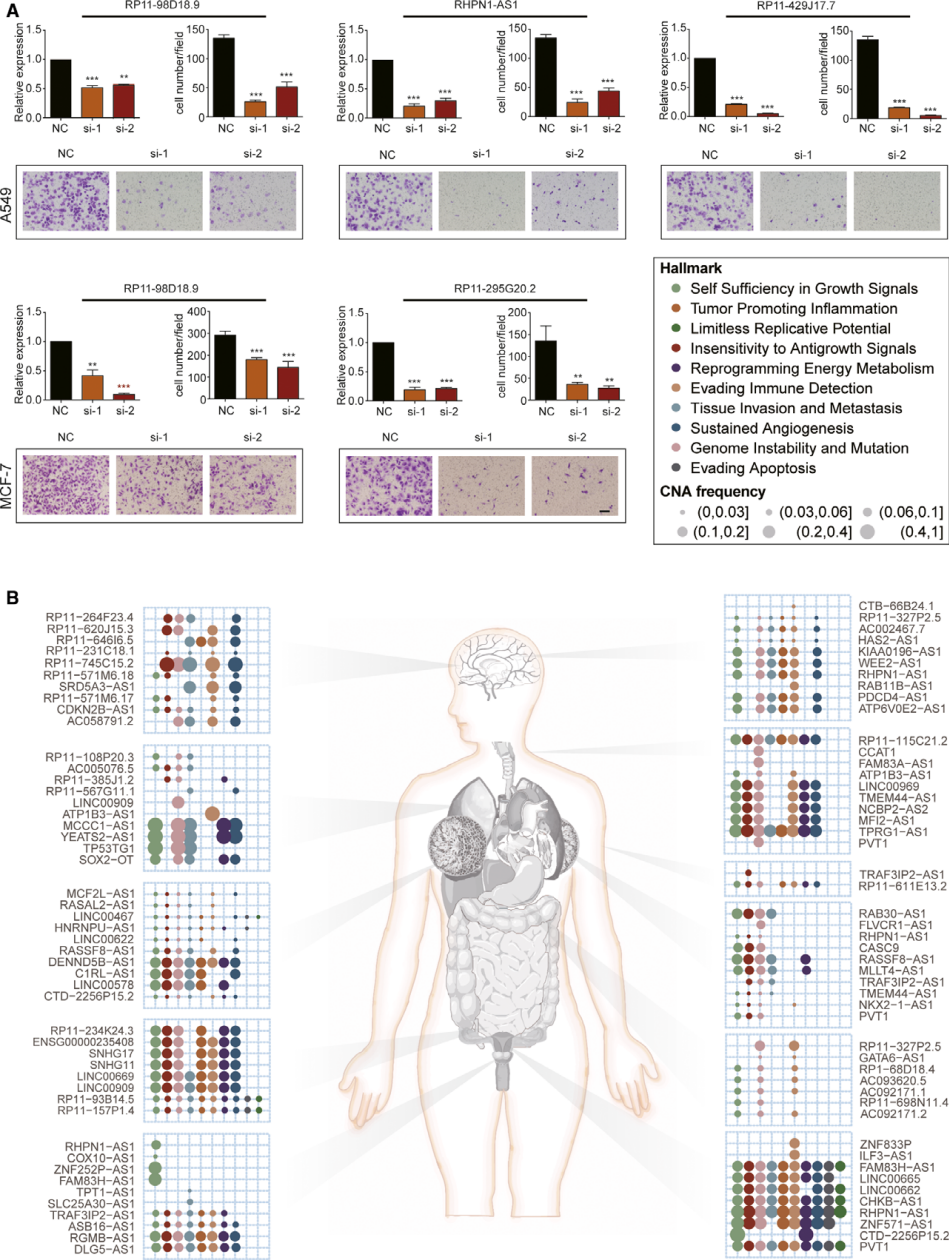
A comprehensive landscape of driver lncRNAs. (A) Validation of lncRNAs that are associated with the hallmark ‘Tissue Invasion and Metastasis’ in A549 and MCF‐7 cell lines, respectively. A quantitative PCR assay was performed to assess the efficiencies of lncRNAs. GAPDH acted as internal control. The Transwell migration assay was performed to assess the migration assay. Cells were fixed and stained with crystal violet. Representative photographs (magnification, 100×) are shown. The number of migration cell was counted. Data are presented as the mean ± SD of three independent experiments (**P <* *0.01, ***P <* *0.001, unpaired Student's *t* test). (B) A comprehensive landscape of hallmark‐associated driver lncRNAs in 11 cancer types. For each cancer type, 10 representative driver lncRNAs are shown. The color and size of the circle indicate cancer hallmarks and CNA frequencies in orresponding cancer type.

Our above observations indicated that these genetically altered lncRNAs may substantially contribute to tumorigenesis by inducing similar functional effects with known cancer driver PCGs in a mutually exclusive manner, suggesting their cancer‐driving roles. As a proof of concept, we validate driver roles of the lncRNAs associated with the hallmark ‘Tissue Invasion and Metastasis’ in lung adenocarcinoma and breast cancer using corresponding A549 and MCF‐7 cell lines, respectively. Indeed, to eleminate inconsistency, candidate driver lncRNAs with inconsistent copy number and/or expression between cancer tissue and cell lines (Zhao *et al*., 2017) were filtered. For the remaining nine lncRNAs, three lncRNAs, *FAM83H‐AS1*,* CASC9* and *PVT1*, have been confirmed to promote cell invasion and metastasis. Finally, three lncRNAs (*RP11‐98D18.9*,* RHPN1‐AS1* and *RP11‐429J17.7*) in lung adenocarcinoma and three lncRNAs (*RP11‐98D18.9*,* RP11‐295G20.2* and *LINC00578*) in breast cancer were evaluated, both containing a common lncRNA *RP11‐98D18.9*. Two independent siRNAs were transfected to avoid off‐target effects, and the efficiencies of lncRNAs depletion were assessed by quantitative PCR. As a result, cell migration was significantly reduced by depletion of five of these lncRNAs in lung adenocarcinoma and breast cancer cell lines, as shown by a Transwell migration assay (*P *<* *0.05, unpaired Student's test) (Fig. 8A).

Taken together, these 378 candidate driver lncRNAs from the cancer hallmark‐associated mutually exclusive modules can serve as an excellent resource for further investigation (Fig. 8B). To facilitate the full exploration for the scientific community, we have deposited these candidate driver lncRNAs and their associated mutually exclusive modules across 11 cancer types in a public resource DriverLncRNA (available at http://biocc.hrbmu.edu.cn/DriverLncRNA). We hope and expect that these data‐sharing efforts will drive the research with respect to tumor mechanisms, as well as promote the identification of promising drug targets and the development of more effective treatment options for the benefit of patients.

## 4 Discussion

In the past decades, the emerging roles of lncRNAs in cancer have broadened our conceptions of tumorigenesis. However, an identification the driver genetic events in lncRNAs that provide fitness advantages and promote clonal extension during cancerogenesis is largely lacking. In the present study, we performed the largest systematic analysis of genetically altered lncRNAs by analyzing more than 5000 samples across 11 cancer types from TCGA. We identified, for the first time, a total of 378 candidate driver lncRNAs based on functional redundancy of mutual exclusivity with known cancer driver PCGs. These candidate driver lncRNAs share a series of genomic features. including evolutionary conservation, early replication and open chromatin accessibility with known cancer driver PCGs. Our study expands the catalog of genetic events implicated in cancer development, opens new avenues for understanding the functional roles of lncRNAs in tumorigenesis, and thus serves as a valuable resource to direct more in‐depth experimentation.

It should be noted that whether driver lncRNAs exist remains a complex controversial issue. According to the definition provided in Vogelstein *et al*. (2013), driver gene mutation can confer selective growth advantage to the cells in which it occurs. Recent accumulating evidence did reveal that several lncRNAs with genetic alterations can confer selective growth advantage, satisfying the definition of drivers. For example, lncRNA *FAL1* amplified in ovarian cancers could promote cell proliferation by recruiting the chromatin repressor protein *BMI‐1* and inhibiting the expression of *CDKN1A* (Hu *et al*., 2014). Focal amplifications of lncRNA *SAMMSON* were observed in approximately 10% of melanomas, which increased the viability of melanoma cells by regulating vital mitochondrial functions (Leucci *et al*., 2016). Also, the concept of ‘driver lncRNAs’ was frequently noted and discussed in recent published reviews (Lin and Yang, 2018; Schmitt and Chang, 2016). Accordingly, we reasoned that driver lncRNAs actually exist. With the accumulation of whole genome sequence data and an understanding of the molecular mechanism of lncRNAs, more driver lncRNAs may be reported in the future.

However, the key to identifying driver lncRNAs comprises making a distinction from passenger lncRNAs and some non‐driver cancer lncRNAs that functionally contribute to cancer phenotypes without driver roles. By definition, driver lncRNAs are able to provide selective growth advantage to cancer cells by contributing to various malignant phenotypes, whereas non‐driver cancer lncRNAs are not. Recurrence is considered as one potential sign of positive selection among tumors (Dees *et al*., 2012; Fu *et al*., 2014); therefore, functional lncRNAs with recurrent genetic alterations are more likely to be driver lncRNAs, instead of non‐driver cancer lncRNAs. With the aim of identifying driver lncRNAs, our method considers lncRNAs that show recurrent copy number alteration, exhibit mutually exclusive patterns with known cancer drivers, and affect various cancer hallmarks. Moreover, the candidate driver lncRNAs identified in the present study shared similar genomic patterns with driver PCGs and could thus serve as an excellent candidate for further investigation using functional and mechanistic experiments.

Somatic copy number variations are one of the major source of genetic alteration that contributes to the neoplastic process (Kim *et al*., 2013; Zack *et al*., 2013). Several studies have revealed some lncRNAs with CNA playing driver roles during tumorigenesis. Driver lncRNA *PVT1* was amplified in 18.8% of pan cancers, and gain of *PVT1* was required for by stabilizing *MYC* protein (Tseng *et al*., 2014). lncRNA *NORAD*, amplified in 8% of colorectal adenocarcinoma, regulated genomic stability by sequestering *PUMILIO* protein (Cerami *et al*., 2012; Lee *et al*., 2016; Tichon *et al*., 2016). Other driver lncRNAs with frequent CNA promoted the growth of cancer cells, including *BCAL8* (Yan *et al*., 2015), and *SOX2OT* (Hou *et al*., 2014; Wu *et al*., 2018a). Importantly, most of these known CNA driver lncRNAs were also identified in the present study, highlighting the efficiency of our methods. CNA driver lncRNA *FAL1* and *SAMMSON* were not identified by our method, and they possibly follow other genomic patterns rather than mutual exclusivity.

Our results demonstrate that lncRNAs underwent numerous genetic alterations and were extensively involved in mutual exclusivity with well‐known cancer driver PCGs. The recent application of massively parallel next‐generation sequencing to a growing number of cancer genomes has revealed abundant mutually exclusive genetic alteration events (Cancer Genome Atlas, 2012; Network, 2013; Ping *et al*., 2014), including numerous known cancer driver genes, such as *RAS* and *TP53*. These mutually exclusive genetic alterations can affect similar downstream pathways, exhibiting strong functional redundancy (Ciriello *et al*., 2012). Such functional redundancy of mutual exclusivity can be explained according to the clonal evolution hypothesis of tumor progression (Gillies *et al*., 2012). When damage of a driver gene is sufficient to disturb the activity of certain key pathways, other gene alterations with similar functional consequences will offer no further selective advantage on that clone; that is the selection pressure on these other alterations could be diminished or even nullified during tumor evolution (Ciriello *et al*., 2012; Remy *et al*., 2015). Therefore, mutual exclusivity is a common and informative phenomenon during cancerogenesis (Deng *et al*., 2017), which has important implications for the functional exploration of lncRNAs in cancer pathogenesis, and helps to uncover novel drivers and decipher their downstream functions.

Notably, using mutual exclusivity, we captured a subset of less frequently altered candidate driver lncRNAs (in < 5% of the samples). Among these, *LINC‐PINT* (amplified in 3.3% samples of GBM), *TRAF3IP2‐AS1* (deleted in 2.8% samples of LUAD) and *TP53TG1* (amplified in 4.0% samples of LUSC), which have been recorded in known cancer lncRNA databases, were nominated for mutual exclusivity with established cancer PCGs, such as *EGFR* for *LINC‐PINT*,* PIP5K1A* for *TRAF3IP2‐AS1* and *BCL2* for *TP53TG1*, suggesting that these infrequent candidate driver lncRNAs harbored functionally redundant genetic lesions with known cancer PCGs and conferred similar selective growth advantage for tumor cells. Hence, identifying candidate driver lncRNAs based on mutual exclusivity has provided additional clues about the mechanisms underlying tumorigenesis, allowing more samples to be covered and explaining previously uncharacterized patients. In addition, 71 candidate driver lncRNAs were predictive of survival in various cancer types. Especially, both amplification and high expression of candidate driver lncRNA *AC000123.4* indicated poor survival in LGG, independent of clinical characteristics and molecular markers.

The complexity of cancer can be simplified into several distinctive and complementary capabilities (‘cancer hallmarks’) that enable tumor growth and metastasis (Hanahan and Weinberg, 2000, 2011). Accumulating evidence has shown that a group of lncRNAs contribute to the cancer hallmarks (Bhan *et al*., 2017; Schmitt and Chang, 2016). In the present study, hallmark‐associated mutually exclusive networks help not only to identify candidate driver lncRNAs, but also to determine their roles in the cancer hallmarks. Generally, all of the 10 hallmarks were affected by several candidate driver lncRNAs, with ‘Genome Instability and Mutation’ for the most numerous candidate driver lncRNAs and ‘Limitless Replicative Potential’ for the least numerous ones. In LUAD, candidate driver lncRNA *CASC9* was associated with the hallmark ‘Self Sufficiency in Growth Signals’, which could be confirmed by the phenomena that *CASC9* promoted lung adenocarcinoma cell proliferation (Zhou *et al*., 2018). In BRCA, *LINC01133* that inhibited epithelial‐mesenchymal transition was found to affect hallmark ‘Tissue Invasion and Metastasis’ (Kong *et al*., 2016). As proof of principle, we successfully validated five out of six candidate driver lncRNAs that were associated with the hallmark ‘Tissue Invasion and Metastasis’ in lung adenocarcinoma and breast cancer. Moreover, GO terms assigned to each hallmark allow us to explore more specific mechanisms. For example, in the hallmark ‘Evading Immune Detection’, we found that GO term ‘regulation of defense response to virus’ was only affected by mutually exclusive modules in HNSC, which is consistent with the prevalence and the roles of human papillomavirus in directly inhibiting innate immune system for this cancer type (Bodily and Laimins, 2011; Maxwell *et al*., 2016). Similarly, GO term ‘neurotransmitter secretion’ in the hallmark ‘Self Sufficiency in Growth Signals’ was unique to GBM and LGG, in which neurotransmitters such as dopamine and *GABA*, have proved to govern cell proliferation (Dolma *et al*., 2016; El‐Habr *et al*., 2017).

The hallmark‐associated driver lncRNAs generated in the present study could facilitate experimental exploration of lncRNAs in cancer pathogenesis. Furthermore, their mutually exclusive partners could provide important implications for our mechanical understanding of cancer lncRNAs. In the results of the present study, driver lncRNA *PVT1* was mutually exclusive with *CDKN2A* in LUAD, and with *CCNE*1 in OV. Both *CCNE1* and *CDKN2A* could contribute to genomic instability by DNA replication stress. A previous study has demonstrated that *PVT1* epigenetically repressed the expression of *CDKN2A* by binding to the *EZH2* (Kong *et al*., 2015); therefore, amplification of *PVT1* and deletion of *CDKN2A* could consistently trigger the expression abnormality of *CDKN2A* and in turn lead to genomic instability. Similarly, amplification of *PVT1* was indispensable for the stability of *MYC* (Tseng *et al*., 2014), which could influence the expression of *CCNE1* (Benaud and Dickson, 2001). Hence, *PVT1* may share downstream effects with the amplification of *CCNE1*. Interestingly, *CCNE1* suffered much more frequent CNA (24.6%) than *CDKN2A* (4.5%) in OV, whereas the opposite trend was observed in LUAD (5.8% and 19.0% for *CCNE1* and *CDKN2A*, respectively), indicating that amplification of *PVT1* may dysregulate different downstream genes, which in turn contribute to cancer development.

Other non‐coding RNAs, such as miRNAs that regulate complementary mRNAs by inducing translational repression and mRNA decay, also play indispensable oncogene roles in tumorigenesis (Iwakawa and Tomari, 2015). Therefore, we systematically examined miRNA located in the genomic regions of candidate driver lncRNAs identified in the present study. As a result, a total of 12 miRNAs were found to be embedded in genomic regions of seven lncRNAs (Table S10). Through comprehensive literature annotation for these miRNAs and lncRNAs, we found that two lncRNAs (*PVT1* and *MIR31HG*) and the embedded five miRNAs (*hsa‐mir‐1204*,* hsa‐mir‐1205*,* hsa‐mir‐1206*,* hsa‐mir‐1207*;* hsa‐mir‐31*) play oncogenic roles that were widely confirmed by previous studies (Guan *et al*., 2007; Kong *et al*., 2015; Liu *et al*., 2018; Shih *et al*., 2017; Valastyan *et al*., 2009; Wu *et al*., 2018b; Yamagishi *et al*., 2012; Yan *et al*., 2017). These findings suggest that both of these lncRNAs and derived miRNAs contribute to tumorigenesis, although, for lncRNAs *LINC00969*,* U47924.29* and *LINC00669*, we did not find evidence of oncogenic roles recorded in literature. By contrast, their derived miRNAs (including *hsa‐mir‐570*,* hsa‐mir‐200c*,* hsa‐mir‐141* and *hsa‐mir‐924*) were reported to play roles in tumorigenesis (Table S10), suggesting that these derived miRNAs, rather than the lncRNAs, are the potential drivers. Interestingly, lncRNA (*RAB11B‐AS1*) was markedly down‐regulated in human osteosarcoma, and was recently confirmed to be associated with proliferation, migration, invasiveness and apoptosis of cell (Chen *et al*., 2018), whereas its derived *hsa‐mir‐4999* was not found to be implicated in any cancer.

## 5 Conclusions

In summary, the present study represents a proof‐of‐principle study for identifying candidate driver lncRNAs through integrative analyses of genomic, transcription datasets and cancer hallmarks. The comprehensive landscape of candidate driver lncRNAs and their constituted cancer hallmark‐associated mutually exclusive modules (freely accessed at the database DriverLncRNA, http://biocc.hrbmu.edu.cn/DriverLncRNA) provide a useful resource for an understanding of cancer mechanisms, which would also lead to clinical benefits in diagnosis, prognosis and treatment, as well as contribute further to personalized medicine.

## Author contributions

XL, YX and XG conceived the project. YX, YLD, SYL, XXZ and CXZ designed the experiment. YX and YLD designed the framework to identify hallmark‐associated mutually exclusive modules. SYL performed module analysis and clinical analysis. XXZ performed copy number analysis and pan‐cancer analysis. CXZ performed lncRNA functional verification. HTY, GML, LWX, YJL, CYD and TTZ provided technical assistance. XL, YX, YLD, SYL and XXZ edited the manuscript. All authors read and approved the final manuscript submitted for final publication.

## Supporting information


**Fig. S1.** The distribution of semantic similarity score between candidate GO terms and hallmark‐associated GO terms.
**Fig. S2.** Heatmaps show enrichment of hallmark‐associated PCGs in additional pathways that were downloaded from Synapse.
**Fig. S3.** Construction of mutually exclusive networks from binary copy number alteration profiles.
**Fig. S4.** The lncRNAs and protein‐coding genes in the top 10 most significant wide peak regions in BRCA, HNSC, LUAD, LUSC, PRAD, LGG, STAD, CR, SKCM and OV.
**Fig. S5.** The basic statistics of mutually exclusive module across 11 major cancer types.
**Fig. S6.** The number of predicted lncRNA from real data and random CNAs profiles and expression profiles across 11 cancer types.
**Fig. S7.** QQ plot of *P*‐values that evaluate the hallmark associated function of mutually exclusive modules.
**Fig. S8.** The overlap of candidate driver genes with Cancer Gene Census.
**Fig. S9.** Proportion of lncRNAs from modules lie within early‐replicating regions.
**Fig. S10.** Heatmaps show cancer‐associated hallmarks affected by cancer‐specific driver lncRNAs.
**Fig. S11.** Heatmap shows cancer‐associated hallmarks affected by PVT1 in HNSC, LUAD and OV.
**Fig. S12.** Kaplan–Meier plots of DFS of LGG patients grouped by copy number status of lncRNA AC000123.4.
**Table S1.** Detailed information of patients analyzed in the present study.
**Table S2.** The number of copy number altered PCGs and lncRNAs across cancers.
**Table S3.** The number of mutually exclusive modules and driver lncRNAs across cancers.
**Table S4.** The overlap of candidate driver lncRNAs identified by MiTranscriptome and our method.
**Table S5.** The overlap of candidate driver lncRNAs identified by CRISPRi and our method.
**Table S6.** Cox proportional hazards models of driver lncRNAs across cancers.
**Table S7.** Univariate and multivariate Cox proportional hazards analysis of disease‐free survival for 429 brain lower grade glioma patients according to AC000123.4 copy number status.
**Table S8.** Univariate and multivariate Cox proportional hazards analysis of disease free survival for 429 brain lower grade glioma patients according to AC000123.4 expression levels.
**Table S9.** Univariate and multivariate Cox proportional hazards analysis of disease‐free survival for 399 brain lower grade glioma patients without EGFR amplification according to AC000123.4 copy number status.
**Table S10.** The miRNAs from miRBase v22 located in the genomic regions of candidate driver lncRNAs.
