# Supplementary material for: A pan‐cancer atlas of cancer hallmark‐associated candidate driver lncRNAs
Source: Mol Oncol. 2018 Oct 2;12(11):1980–2005. doi: 10.1002/1878-0261.12381 (PMC6210054; doi:10.1002/1878-0261.12381)

Supplementary Tables

Table S1 The detailed information of patients analyzed in manuscript.

Cancer type	Abrr	Gene expression from TCGA Sizes	Copy number variation from TCGA Sizes	LncRNA expression from TANRIC Sizes	Clinical data from cBioPortal Sizes
Stomach adenocarcinoma	STAD	415	441	285	443
Prostate adenocarcinoma	PRAD	297	331	374	499
Skin cutaneous melanoma	SKCM	254	469	226	478
Brain lower grade glioma	LGG	469	463	486	530
Breast invasive carcinoma	BRCA	1102	1080	837	1105
Ovarian serous cystadenocarcinoma	OV	266	572	412	607
Lung squamous cell carcinoma	LUAD	490	494	488	522
Lung adenocarcinoma	LUSC	490	490	220	504
Head and neck squamous cell carcinoma	HNSC	451	452	426	530
Glioblastoma multiforme	GBM	169	535	154	607
Colon adenocarcinoma	COAD	192	429	157	453
Rectal adenocarcinoma	READ	72	162	71	162

(As described in previously TCGA studies, COAD and READ were merged and formed one cancer sample set COAD/READ (CR) in subsequent analysis.)

Table S2. The number of copy number altered PCGs and lncRNAs across cancers.

Cancer Type	PCG(amp)	PCG(del)	lncRNA(amp)	lncRNA(del)
BRCA	1914	211	293	20
GBM	99	15	25	9
HNSC	945	168	145	19
LUAD	1760	428	267	51
LUSC	1613	170	283	33
PRAD	104	969	35	210
LGG	507	421	134	125
STAD	1236	156	147	13

CR	545	141	34	8
SKCM	775	190	135	29
OV	3075	228	496	29

amp, amplification; del, deletion

Table S3 The number of mutually exclusive modules and driver lncRNAs across cancers.

Abbreviation	Sample sizes	Candidate lncRNA number	Driver lncRNA number	Module number
STAD	441	160	7	15
PRAD	331	245	45	60
SKCM	469	164	2	6
LGG	463	259	41	35
BRCA	1080	313	88	282
OV	572	525	133	1547
LUAD	494	318	69	379
LUSC	490	316	48	97
HNSC	452	164	27	55
GBM	535	34	14	54
CR	591	42	9	25

Table S4 The overlap of candidate driver lncRNAs identified by MiTranscriptome and our method.

Cancer types	MiTranscriptome	Our results	Overlap	P value
HNSC	382	27	3	3.72e-02
LUSC	497	48	9	4.37e-05
LUAD	481	69	7	9.76e-03
PRAD	412	45	6	2.02e-03
STAD	64	7	1	3.19e-02
BRCA	521	88	5	2.36e-01
Total	1577	238	71	6.28e-15

Table S5 The overlap of candidate driver lncRNAs identified by CRISPRi and our method.

Cancer types	CRISPRi	Our results	Overlap	P value
BRCA	55	88	3	5.10e-03
GBM	37	14	0	1.0e-00
Total	286	378	27	1.97e-08

Table S6 Cox proportional hazards models of driver lncRNAs across cancers. # indicated not significant.

Overall Survival Analysis				
	Univariate Analysis		Multivariate Analysis	
Ensembl ID	P value	Hazard ratio	P value	Hazard ratio
head and neck squamous cell carcinoma				
ENSG00000246889	2.50E-02	1.11	3.50E-02	1.11
ENSG00000254604	2.90E-02	1.11	3.80E-02	1.10
ENSG00000254605	1.90E-02	1.12	3.00E-02	1.11
lung adenocarcinoma				
ENSG00000246695	2.60E-02	1.36	#	#
ENSG00000256894	1.90E-02	1.37	4.40E-02	1.33
ovarian serous cystadenocarcinoma				
ENSG00000254024	1.07E-02	1.18	7.18E-03	1.19
ENSG00000226686	1.89E-04	1.26	1.70E-03	1.22
ENSG00000267254	4.66E-03	1.20	2.08E-02	1.16
ENSG00000267470	2.30E-03	1.19	9.57E-03	1.16
ENSG00000246263	7.42E-03	1.20	6.70E-03	1.20
ENSG00000236144	1.38E-03	1.23	2.45E-03	1.22
ENSG00000247081	1.76E-03	1.23	9.50E-04	1.24
ENSG00000260708	2.21E-02	1.31	3.77E-02	1.28
ENSG00000267014	5.05E-03	1.16	1.82E-02	1.13
ENSG00000225975	2.87E-03	1.21	1.18E-02	1.18
ENSG00000261824	8.56E-04	1.24	5.96E-03	1.19
ENSG00000267213	1.44E-02	1.15	1.33E-02	1.15
ENSG00000266935	1.47E-03	1.22	6.63E-03	1.19
ENSG00000267053	2.95E-03	1.22	1.00E-02	1.20
ENSG00000267439	3.95E-05	1.31	1.15E-04	1.29

ENSG00000267575	3.59E-04	1.24	3.49E-03	1.19
ENSG00000232677	2.95E-03	1.22	1.00E-02	1.20
ENSG00000180458	2.99E-03	1.19	1.23E-02	1.16
ENSG00000233527	3.66E-03	1.20	1.48E-02	1.17
ENSG00000251136	3.51E-04	1.32	7.56E-05	1.36
ENSG00000260032	4.77E-03	1.26	#	#
ENSG00000270127	4.47E-03	0.76	3.41E-02	0.81
ENSG00000257084	3.69E-02	1.16	#	#
ENSG00000261437	1.10E-03	1.27	7.17E-04	1.29
ENSG00000205885	4.63E-02	1.15	#	#
ENSG00000253217	2.13E-02	1.16	1.17E-02	1.18
ENSG00000256967	5.00E-02	1.15	#	#
ENSG00000253854	1.52E-03	1.28	1.19E-03	1.28
ENSG00000259424	7.55E-03	0.78	#	#
ENSG00000259583	7.63E-03	0.78	4.77E-02	0.83
ENSG00000232386	7.63E-03	0.78	4.77E-02	0.83
ENSG00000267381	3.46E-03	1.18	1.43E-02	1.15
ENSG00000266916	3.13E-03	1.19	1.33E-02	1.16
ENSG00000250899	3.09E-02	1.16	#	#
ENSG00000177406	4.75E-02	1.14	#	#
ENSG00000266930	1.01E-02	1.16	9.88E-03	1.16
ENSG00000253878	4.16E-03	1.24	3.46E-03	1.25
breast invasive carcinoma				
ENSG00000228801	3.38E-02	1.27	#	#
ENSG00000251136	1.15E-02	1.29	#	#
brain lower grade glioma				
ENSG00000241764	9.74E-03	1.74	#	#
ENSG00000231856	3.22E-03	0.39	4.59E-02	0.46
ENSG00000217576	3.19E-03	0.39	4.54E-02	0.46
ENSG00000234418	3.51E-02	1.55	#	#
ENSG00000228031	3.18E-02	1.52	#	#
ENSG00000238099	6.57E-03	0.39	#	#
ENSG00000260917	3.33E-16	0.12	1.55E-09	0.16
ENSG00000203497	1.11E-16	0.11	1.02E-07	0.18
ENSG00000267289	4.37E-02	2.66	#	#
ENSG00000224138	1.75E-02	1.64	#	#
ENSG00000269386	4.24E-02	2.67	#	#
ENSG00000225559	4.42E-02	1.48	#	#
ENSG00000227869	9.61E-03	1.75	#	#
lung squamous cell carcinoma				
ENSG00000224046	2.38E-02	0.66	2.28E-02	0.64
ENSG00000182165	2.64E-02	0.67	2.57E-02	0.65

ENSG00000253414	9.07E-03	0.77	5.29E-03	0.76
ENSG00000264868	1.40E-02	0.65	1.58E-02	0.64
ENSG00000232019	1.88E-02	0.66	2.17E-02	0.65
ENSG00000242687	2.82E-02	0.68	2.94E-02	0.67
glioblastoma multiforme				
ENSG00000234707	1.15E-02	1.08	#	#

Disease Free Survival Analysis

	Univariate Analysis		Multivariate Analysis	
Ensembl ID	P value	Hazard ratio	P value	Hazard ratio
head and neck squamous cell carcinoma				
ENSG00000255310	3.80E-02	0.57	2.50E-02	0.53
ENSG00000269918	3.80E-02	0.57	2.50E-02	0.53
ENSG00000246089	2.70E-02	0.54	1.70E-02	0.51
ovarian serous cystadenocarcinoma				
ENSG00000247081	3.20E-03	1.21	9.31E-03	1.18
ENSG00000254353	8.50E-03	1.47	1.45E-02	1.44
ENSG00000267213	4.46E-02	1.13	1.45E-02	1.16
ENSG00000269983	8.50E-03	1.47	1.45E-02	1.44
ENSG00000251136	2.70E-03	1.26	3.73E-03	1.24
ENSG00000260032	2.54E-04	1.36	7.63E-03	1.27
ENSG00000270127	1.88E-02	0.81	#	#
ENSG00000270017	3.55E-02	0.82	#	#
ENSG00000261437	5.26E-03	1.23	1.60E-02	1.19
ENSG00000253854	1.06E-02	1.22	2.65E-02	1.18
ENSG00000259424	1.91E-02	0.81	#	#
ENSG00000259583	3.51E-02	0.83	#	#
ENSG00000232386	3.51E-02	0.83	#	#
ENSG00000242622	4.04E-02	1.24	#	#
ENSG00000232453	2.40E-02	1.33	1.86E-02	1.35
ENSG00000253878	3.02E-02	1.17	#	#
brain lower grade glioma				
ENSG00000241764	8.38E-06	2.15	3.37E-04	1.95
ENSG00000234418	4.81E-06	2.20	1.96E-04	2.01
ENSG00000228031	1.14E-05	2.11	7.00E-04	1.89
ENSG00000238099	6.43E-03	0.41	#	#
ENSG00000204934	9.09E-06	2.17	1.33E-04	2.05
ENSG00000260917	2.21E-11	0.20	7.94E-08	0.24
ENSG00000261342	3.04E-02	2.45	#	#
ENSG00000203497	0.00E+00	0.13	5.51E-11	0.17
ENSG00000267299	3.99E-02	2.54	#	#

ENSG00000267289	3.42E-02	2.61	#	#
ENSG00000270157	1.35E-05	2.10	4.50E-04	1.93
ENSG00000224138	2.94E-06	2.24	2.64E-04	1.99
ENSG00000269386	3.78E-02	2.57	#	#
ENSG00000261455	4.74E-06	2.21	1.45E-04	2.04
ENSG00000182648	2.60E-06	2.23	#	#
ENSG00000228775	1.35E-05	2.10	4.50E-04	1.93
ENSG00000225559	6.75E-06	2.14	4.37E-04	1.93
ENSG00000240449	9.09E-06	2.17	1.33E-04	2.05
ENSG00000227869	8.18E-06	2.18	3.78E-04	1.96
ENSG00000260231	1.38E-05	2.10	4.72E-04	1.93
ENSG00000239377	9.09E-06	2.17	1.33E-04	2.05
lung squamous cell carcinoma				
ENSG00000253414	8.37E-03	0.71	3.52E-03	0.68
ENSG00000265943	2.10E-04	1.89	5.26E-04	1.85
ENSG00000266729	6.75E-03	1.64	6.10E-03	1.66
ENSG00000246889	2.32E-02	1.18	#	#
ENSG00000244124	1.78E-02	0.71	2.57E-02	0.72
ENSG00000248932	5.72E-03	0.64	8.98E-03	0.65

Table S7 Univariate and multivariate Cox proportional hazards analysis of disease free survival for 429 brain lower grade glioma patients according to AC000123.4 copy number status.

Subgroup	Univariate Analysis		Multivariate Analysis	
	Hazard ratio (95% CI)	P value	Hazard ratio (95% CI)	P value
All patients (N=429)				
Age, modeled as a continuous variable	1.02(1.01-1.04)	1.52e-03	1.02(1.01-1.03)	7.43e-03
Gender				
Male vs. Female	0.85(0.61-1.20)	0.36	0.84(0.60-1.19)	0.33
Histological diagnosis				
Oligoastrocytoma vs. Astrocytoma	0.56(0.35-0.89)	1.35e-02	0.56(0.35-0.88)	1.32e-02
Oligodendroglioma vs. Astrocytoma	0.64(0.44-0.94)	2.15e-02	0.66(0.45-0.99)	4.39e-02

Grade				
G3 vs. G2	1.55(1.10-2.18)	1.31e-02	1.40(0.98-2.00)	6.76e-02
Continuous copy number				
AC000123.4	2.12(1.49-3.02)	3.06e-05	1.99(1.38-2.88)	2.44e-04

(Abbreviations: CI, confidence interval.)

Table S8 Univariate and multivariate Cox proportional hazards analysis of disease free survival for 429 brain lower grade glioma patients according to AC000123.4 expression levels.

Subgroup	Univariate Analysis		Multivariate Analysis	
	Hazard ratio (95% CI)	P value	Hazard ratio (95% CI)	P value
All patients (N=429)				
Age, modeled as a continuous variable	1.02(1.01-1.04)	1.42e-03	1.02(1.01-1.04)	9.76e-04
Gender				
Male vs. Female	0.84(0.60-1.18)	0.32	0.83(0.59-1.17)	0.29
Histological diagnosis				
Oligoastrocytoma vs. Astrocytoma	0.57(0.36-0.90)	1.58e-02	0.71(0.44-1.14)	0.15
Oligodendroglioma vs. Astrocytoma	0.65(0.45-0.95)	2.73e-02	0.73(0.48-1.10)	0.13
Grade				
G3 vs. G2	1.54(1.09-2.17)	1.47e-02	1.30(0.90-1.87)	0.16
Continuous expression				
AC000123.4	4.41(2.46-7.80)	5.92e-07	3.46(1.92-6.22)	3.44e-05

(Abbreviations: CI, confidence interval.)

Table S9 Univariate and multivariate Cox proportional hazards analysis of disease free survival for 399 brain lower grade glioma patients without EGFR amplification according to AC000123.4 copy number status.

Subgroup	Univariate Analysis	Multivariate Analysis

	Hazard ratio (95% CI)	P value	Hazard ratio (95% CI)	P value
All patients (N=399)				
Age, modeled as a continuous variable	1.01(1-1.02)	0.04	1.01(0.99-1.02)	0.06
Gender				
Male vs. Female	0.84(0.59-1.19)	0.34	0.83(0.59-1.18)	0.30
Histological diagnosis				
Oligoastrocytoma vs. Astrocytoma	0.51 (0.31-0.83)	0.007	0.47(0.29-0.77)	0.002
Oligodendroglioma vs. Astrocytoma	0.70(0.48-1.03)	0.071	0.65(0.44-0.96)	0.03
Continuous copy number				
AC000123.4	1.86(1.26-2.76)	0.0018	1.91 (1.27-2.86)	0.0017

(Abbreviations: CI, confidence interval.)

Table S10 The miRNAs from miRBase v22 that located in the genomic regions of candidate driver lncRNAs.

Pri-miRNA	miRNA	Driver miRNA evidence	LncRNA name	Driver lncRNA evidence	Cancer type
hsa-mir-31	hsa-miR-31-3p, hsa-miR-31-5p	PMID: 22264793; 19524507; 29051494; 24582497	MIR31HG	PMID:25908244; 28639619; 27334046; 26549028	LUSC
hsa-mir-570	hsa-miR-570-3p, hsa-miR-570-5p	PMID:29874097;26084609; 29795113	LINC00969		HNSC
hsa-mir-1204	hsa-mir-1204	PMID:29555976;25756509; 22110125; 18314482	PVT1	PMID:26481356;23175614; 25043044;29752292;29731168	HNSC, LUAD, OV
hsa-mir-1205	hsa-mir-1205	PMID:29852168; doi:10.1158/1538-7445.AM2015-187	PVT1	PMID:26481356;23175614; 25043044;29752292;29731168	HNSC, LUAD, OV
hsa-mir-1206	hsa-miR-1206	PMID:28628559;23077621	PVT1	PMID:26481356;23175614;	HNSC, LUAD, OV

				25043044;29752292;29731168	
hsa-mir-1207	hsa-miR-1207-3p,	PMID:28235236;26337084;	PVT1	PMID:26481356;23175614;	HNSC, LUAD, OV
	hsa-miR-1207-5p	29387230; 27852979		25043044;29752292;29731168	
hsa-mir-200c	hsa-miR-200c-3p,	PMID:20579395;17804704;	U47924.29		OV,BRCA
	hsa-miR-200c-5p	28436968; 19665978			
hsa-mir-141	hsa-miR-141-3p,	PMID:24285464;22479552;	U47924.29		OV,BRCA
	hsa-miR-141-5p	24000293; 28112170			
hsa-mir-924	hsa-miR-924	PMID:29753758;27525335	LINC00669		CR
hsa-mir-5583-2	hsa-miR-5583-5p,		LINC00669		CR
	hsa-miR-5583-3p				
hsa-mir-24-2	hsa-miR-24-3p,	PMID:25943634;27625395;	MIR24-2		OV
	hsa-miR-24-2-5p	22911661; 21463514			
hsa-mir-4999	Has-miR-4999-5p,		RAB11B-AS1	PMID: 29928484	LGG
	hsa-miR-4999-3p				

Supplementary Figure legends

Figure S1 The distribution of semantic similarity score between candidate GO terms and hallmark-associated GO terms. Red arrows indicate Rest-similarity score that was calculated between each curated GO term of a certain hallmark and the rest of other curated GO terms of this hallmark.

Figure S2 Heatmaps show enrichment of hallmark-associated PCGs in additional pathways that were downloaded from Synapse (syn1741407). The P-value were calculated using the hypergeometric test.

Figure S3 Construction of mutually exclusive networks from binary copy number alteration profiles. For each pair of genes, a 2x2 contingency table was built to investigate their relationship of CAN. Gene pair g1 and g2 were significantly mutually exclusive by a hypergeometric test ($p < 0.05$). The co-occurrence of gene pair g3 and g4 was never observed in any samples.

Figure S4 The lncRNAs and protein-coding genes in the top 10 most significant wide peak regions in BRCA, HNSC, LUAD, LUSC, PRAD, LGG, STAD, CR, SKCM and OV. The numbers of protein-coding genes (left) and lncRNAs (right) in each peak are indicated in parentheses. Pie chart of each peak shows the proportion of PCG (blue) and lncRNA (green).

Figure S5 The basic statistics of mutually exclusive module across 11 major cancer types. (A)Stacked bars indicate the distribution of module size. (B) Box plot shows frequencies of CNA for PCGs from hallmark-associated mutually exclusive modules (left) and other PCGs (right) (*: $p < 0.05$, permutation test).

Figure S6 The number of predicted lncRNA from real data (red arrow) and random CNAs profiles (left) and expression profiles (right) across 11 cancer types (curve) (*: $p < 0.05$, permutation test).

Figure S7 QQ-plot of p-values that evaluate the hallmark associated function of mutually exclusive modules. The proportion of p-values are shown in the bottom.

Figure S8 The overlap of candidate driver genes with Cancer Gene Census (CGC). Statistical significance is assessed by the hypergeometric test with $p < 0.05$.

Figure S9 Proportion of lncRNAs from modules lie within early-replicating regions. (A-C) Bar plots show the difference of the fraction of lncRNAs from modules and other lncRNAs that located in early replicating regions derived from all of the 8 cell lines, IMR90 and MCF-7, respectively. The ME lncRNAs in figures indicate lncRNAs from mutually exclusive modules.

Figure S10 Heatmaps show cancer-associated hallmarks affected by cancer-specific driver lncRNAs.

Figure S11 Heatmap shows cancer-associated hallmarks affected by PVT1 in HNSC, LUAD and OV.

Figure S12 Kaplan–Meier plots of DFS of LGG patients grouped by copy number status of lncRNA AC000123.4. The newly added clinical data was downloaded on 1 July, 2016 from cBioPortal website that includes 35 disease-free patients and 11 disease-progressed patients.

Supplementary Figures

Figure S1

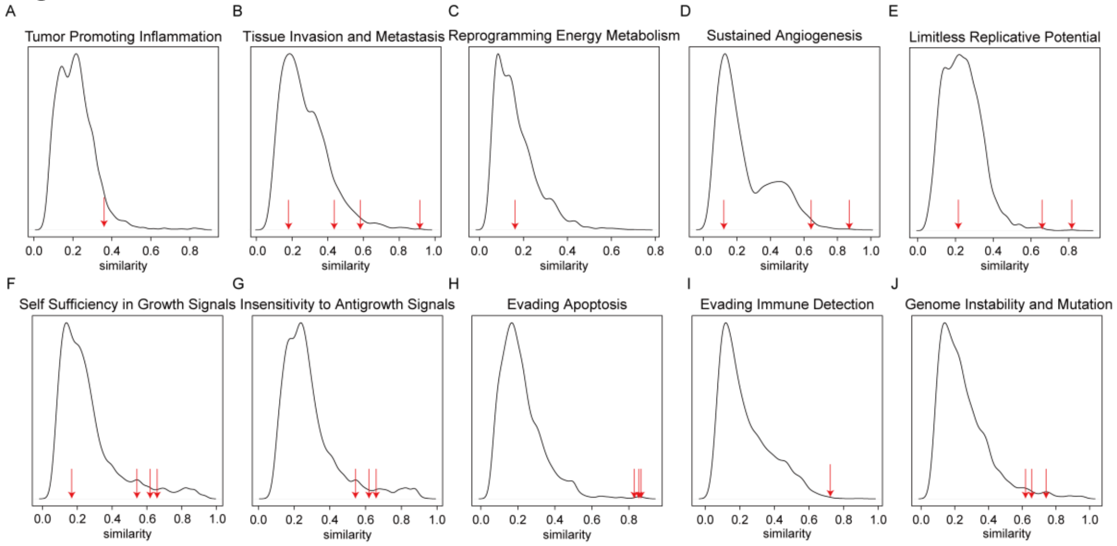

Figure S2

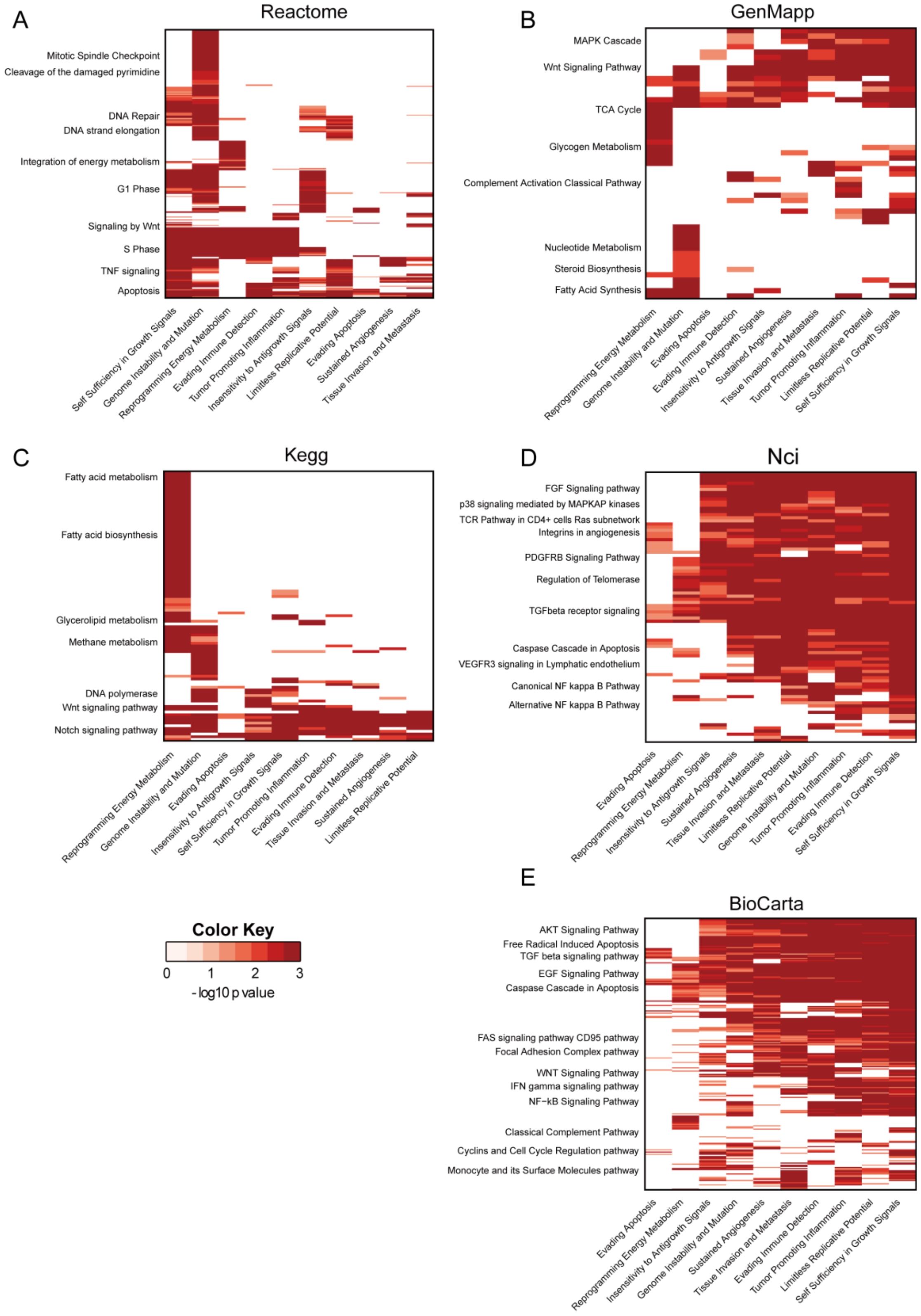
A
Reactome
Mitotic Spindle Checkpoint
Cleavage of the damaged pyrimidine
DNA Repair
DNA strand elongation
Integration of energy metabolism
G1 Phase
Signaling by Wnt
S Phase
TNF signaling
Apoptosis
Self Sufficiency in Growth Signals
Genome Instability and Mutation
Reprogramming Energy Metabolism
Evading Immune Detection
Tumor Promoting Inflammation
Insensitivity to Antigrowth Signals
Limitless Replicative Potential
Evading Apoptosis
Sustained Angiogenesis
Tissue Invasion and Metastasis
B
GenMapp
MAPK Cascade
Wnt Signaling Pathway
TCA Cycle
Glycogen Metabolism
Complement Activation Classical Pathway
Nucleotide Metabolism
Steroid Biosynthesis
Fatty Acid Synthesis
Reprogramming Energy Metabolism
Genome Instability and Mutation
Evading Apoptosis
Evading Immune Detection
Insensitivity to Antigrowth Signals
Sustained Angiogenesis
Tissue Invasion and Metastasis
Tumor Promoting Inflammation
Limitless Replicative Potential
Self Sufficiency in Growth Signals
C
Kegg
Fatty acid metabolism
Fatty acid biosynthesis
Glycerolipid metabolism
Methane metabolism
DNA polymerase
Wnt signaling pathway
Notch signaling pathway
Reprogramming Energy Metabolism
Genome Instability and Mutation
Evading Apoptosis
Insensitivity to Antigrowth Signals
Self Sufficiency in Growth Signals
Tumor Promoting Inflammation
Evading Immune Detection
Tissue Invasion and Metastasis
Sustained Angiogenesis
Limitless Replicative Potential
D
Nci
FGF Signaling pathway
p38 signaling mediated by MAPKAP kinases
TCR Pathway in CD4+ cells Ras subnetwork
Integrins in angiogenesis
PDGFRB Signaling Pathway
Regulation of Telomerase
TGFbeta receptor signaling
Caspase Cascade in Apoptosis
VEGFR3 signaling in Lymphatic endothelium
Canonical NF kappa B Pathway
Alternative NF kappa B Pathway
Evading Apoptosis
Reprogramming Energy Metabolism
Insensitivity to Antigrowth Signals
Sustained Angiogenesis
Tissue Invasion and Metastasis
Limitless Replicative Potential
Genome Instability and Mutation
Tumor Promoting Inflammation
Evading Immune Detection
Self Sufficiency in Growth Signals
Color Key
0
1
2
3
- log10 p value
E
BioCarta
AKT Signaling Pathway
Free Radical Induced Apoptosis
TGF beta signaling pathway
EGF Signaling Pathway
Caspase Cascade in Apoptosis
FAS signaling pathway CD95 pathway
Focal Adhesion Complex pathway
WNT Signaling Pathway
IFN gamma signaling pathway
NF-kB Signaling Pathway
Classical Complement Pathway
Cyclins and Cell Cycle Regulation pathway
Monocyte and its Surface Molecules pathway
Evading Apoptosis
Reprogramming Energy Metabolism
Insensitivity to Antigrowth Signals
Genome Instability and Mutation
Sustained Angiogenesis
Tissue Invasion and Metastasis
Evading Immune Detection
Tumor Promoting Inflammation
Limitless Replicative Potential
Self Sufficiency in Growth Signals

Figure S3

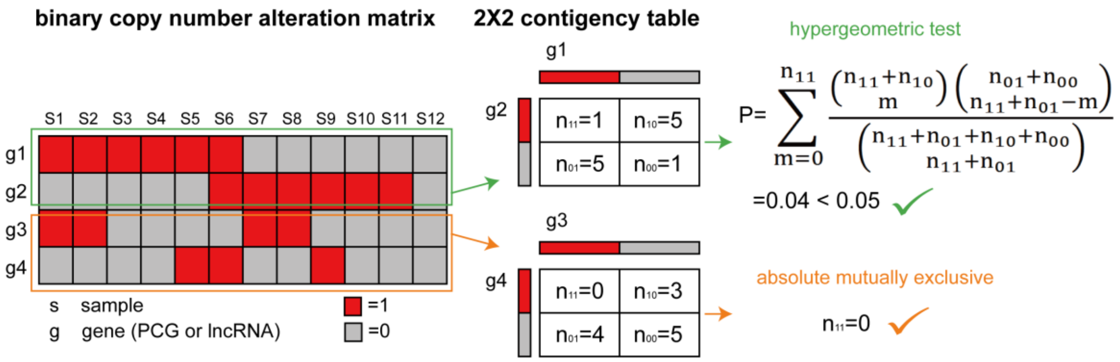

binary copy number alteration matrix
2X2 contigency table
hypergeometric test
S1 S2 S3 S4 S5 S6 S7 S8 S9 S10 S11 S12
g1
g2
g3
g4
s sample
g gene (PCG or lncRNA)
=1
=0
n11=1 n10=5
n01=5 n00=1
P=
=0.04 < 0.05
n11=0 n10=3
n01=4 n00=5
absolute mutually exclusive
n11=0

Figure S4

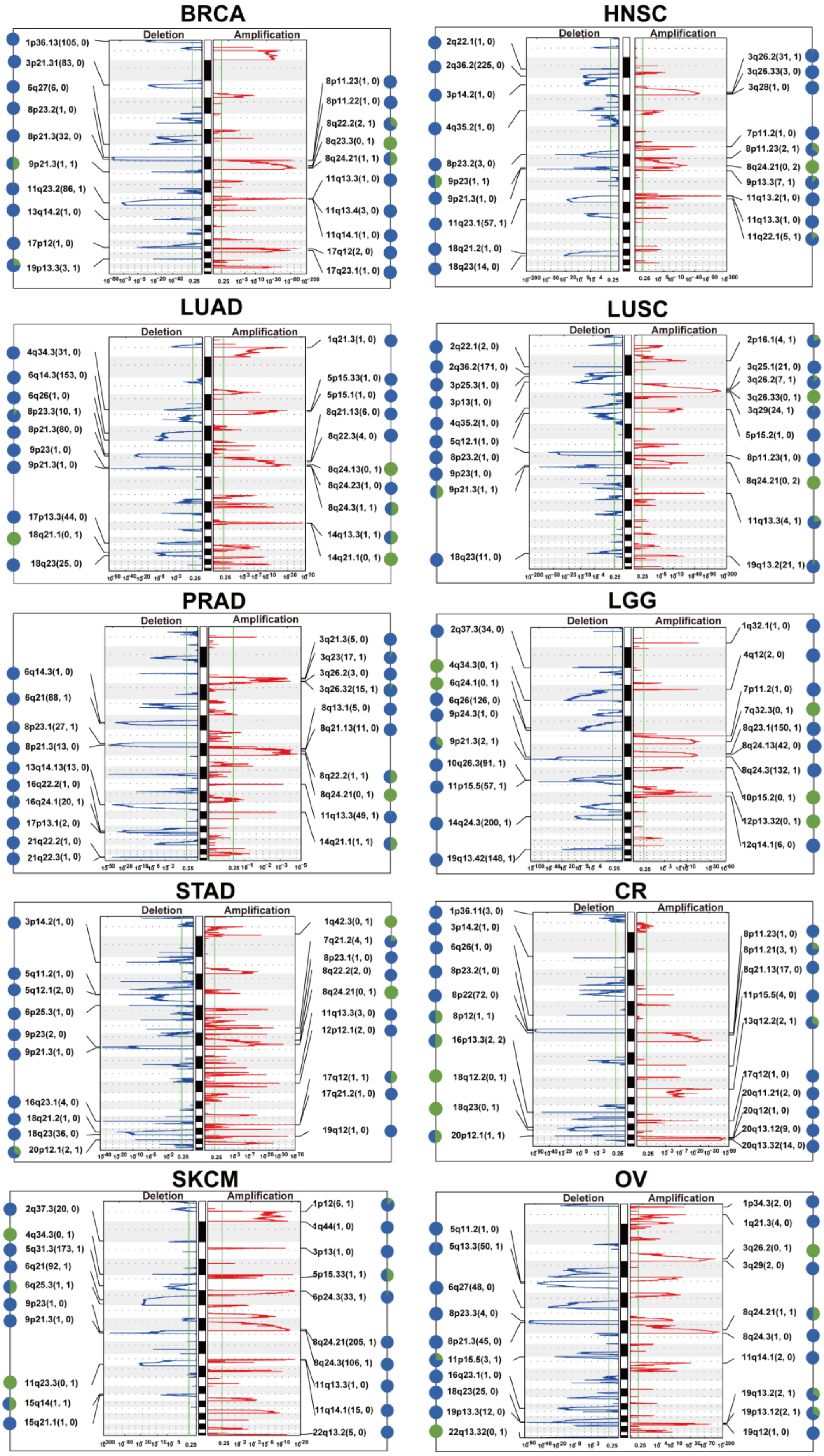
BRCA
Deletion
Amplification
1p36.13(105, 0)
3p21.31(83, 0)
6q27(6, 0)
8p23.2(1, 0)
8p21.3(32, 0)
9p21.3(1, 1)
11q23.2(86, 1)
13q14.2(1, 0)
17p12(1, 0)
19p13.3(3, 1)
8p11.23(1, 0)
8p11.22(1, 0)
8q22.2(2, 1)
8q23.3(0, 1)
8q24.21(1, 1)
11q13.3(1, 0)
11q13.4(3, 0)
11q14.1(1, 0)
17q12(2, 0)
17q23.1(1, 0)
HNSC
Deletion
Amplification
2q22.1(1, 0)
2q36.2(225, 0)
3p14.2(1, 0)
4q35.2(1, 0)
8p23.2(3, 0)
9p23(1, 1)
9p21.3(1, 0)
11q23.1(57, 1)
18q21.2(1, 0)
18q23(14, 0)
3q26.2(31, 1)
3q26.33(3, 0)
3q28(1, 0)
7p11.2(1, 0)
8p11.23(2, 1)
8q24.21(0, 2)
9p13.3(7, 1)
11q13.2(1, 0)
11q13.3(1, 0)
11q22.1(5, 1)
LUAD
Deletion
Amplification
4q34.3(31, 0)
6q14.3(153, 0)
6q26(1, 0)
8p23.3(10, 1)
8p21.3(80, 0)
9p23(1, 0)
9p21.3(1, 0)
17p13.3(44, 0)
18q21.1(0, 1)
18q23(25, 0)
1q21.3(1, 0)
5p15.33(1, 0)
5p15.1(1, 0)
8q21.13(6, 0)
8q22.3(4, 0)
8q24.13(0, 1)
8q24.23(1, 0)
8q24.3(1, 1)
14q13.3(1, 1)
14q21.1(0, 1)
LUSC
Deletion
Amplification
2q22.1(2, 0)
2q36.2(171, 0)
3p25.3(1, 0)
3p13(1, 0)
4q35.2(1, 0)
5q12.1(1, 0)
8p23.2(1, 0)
9p23(1, 0)
9p21.3(1, 1)
18q23(11, 0)
2p16.1(4, 1)
3q25.1(21, 0)
3q26.2(7, 1)
3q26.33(0, 1)
3q29(24, 1)
5p15.2(1, 0)
8p11.23(1, 0)
8q24.21(0, 2)
11q13.3(4, 1)
19q13.2(21, 1)
PRAD
Deletion
Amplification
6q14.3(1, 0)
6q21(88, 1)
8p23.1(27, 1)
8p21.3(13, 0)
13q14.13(13, 0)
16q22.2(1, 0)
16q24.1(20, 1)
17p13.1(2, 0)
21q22.2(1, 0)
21q22.3(1, 0)
3q21.3(5, 0)
3q23(17, 1)
3q26.2(3, 0)
3q26.32(15, 1)
8q13.1(5, 0)
8q21.13(11, 0)
8q22.2(1, 1)
8q24.21(0, 1)
11q13.3(49, 1)
14q21.1(1, 1)
LGG
Deletion
Amplification
2q37.3(34, 0)
4q34.3(0, 1)
6q24.1(0, 1)
6q26(126, 0)
9p24.3(1, 0)
9p21.3(2, 1)
10q26.3(91, 1)
11p15.5(57, 1)
14q24.3(200, 1)
19q13.42(148, 1)
1q32.1(1, 0)
4q12(2, 0)
7p11.2(1, 0)
7q32.3(0, 1)
8q23.1(150, 1)
8q24.13(42, 0)
8q24.3(132, 1)
10p15.2(0, 1)
12p13.32(0, 1)
12q14.1(6, 0)
STAD
Deletion
Amplification
3p14.2(1, 0)
5q11.2(1, 0)
5q12.1(2, 0)
6p25.3(1, 0)
9p23(2, 0)
9p21.3(1, 0)
16q23.1(4, 0)
18q21.2(1, 0)
18q23(36, 0)
20p12.1(2, 1)
1q42.3(0, 1)
7q21.2(4, 1)
8p23.1(1, 0)
8q22.2(2, 0)
8q24.21(0, 1)
11q13.3(3, 0)
12p12.1(2, 0)
17q12(1, 1)
17q21.2(1, 0)
19q12(1, 0)
CR
Deletion
Amplification
1p36.11(3, 0)
3p14.2(1, 0)
6q26(1, 0)
8p23.2(1, 0)
8p22(72, 0)
8p12(1, 1)
16p13.3(2, 2)
18q12.2(0, 1)
18q23(0, 1)
20p12.1(1, 1)
8p11.23(1, 0)
8p11.21(3, 1)
8q21.13(17, 0)
11p15.5(4, 0)
13q12.2(2, 1)
17q12(1, 0)
20q11.21(2, 0)
20q12(1, 0)
20q13.12(9, 0)
20q13.32(14, 0)
SKCM
Deletion
Amplification
2q37.3(20, 0)
4q34.3(0, 1)
5q31.3(173, 1)
6q21(92, 1)
6q25.3(1, 1)
9p23(1, 0)
9p21.3(1, 0)
11q23.3(0, 1)
15q14(1, 1)
15q21.1(1, 0)
1p12(6, 1)
1q44(1, 0)
3p13(1, 0)
5p15.33(1, 1)
6p24.3(33, 1)
8q24.21(205, 1)
8q24.3(106, 1)
11q13.3(1, 0)
11q14.1(15, 0)
22q13.2(5, 0)
OV
Deletion
Amplification
5q11.2(1, 0)
5q13.3(50, 1)
6q27(48, 0)
8p23.3(4, 0)
8p21.3(45, 0)
11p15.5(3, 1)
16q23.1(1, 0)
18q23(25, 0)
19p13.3(12, 0)
22q13.32(0, 1)
1p34.3(2, 0)
1q21.3(4, 0)
3q26.2(0, 1)
3q29(2, 0)
8q24.21(1, 1)
8q24.3(1, 0)
11q14.1(2, 0)
19q13.2(2, 1)
19p13.12(2, 1)
19q12(1, 0)

Figure S5

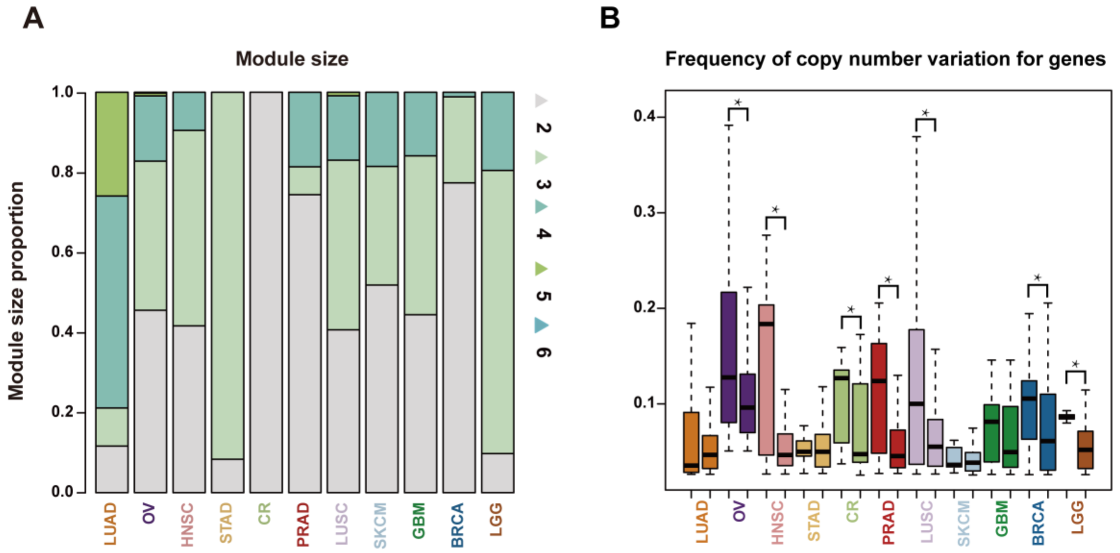

A
Module size
Module size proportion
1.0
0.8
0.6
0.4
0.2
0.0
2
3
4
5
6
LUAD
OV
HNSC
STAD
CR
PRAD
LUSC
SKCM
GBM
BRCA
LGG
B
Frequency of copy number variation for genes
0.4
0.3
0.2
0.1
LUAD
OV
HNSC
STAD
CR
PRAD
LUSC
SKCM
GBM
BRCA
LGG

Figure S6

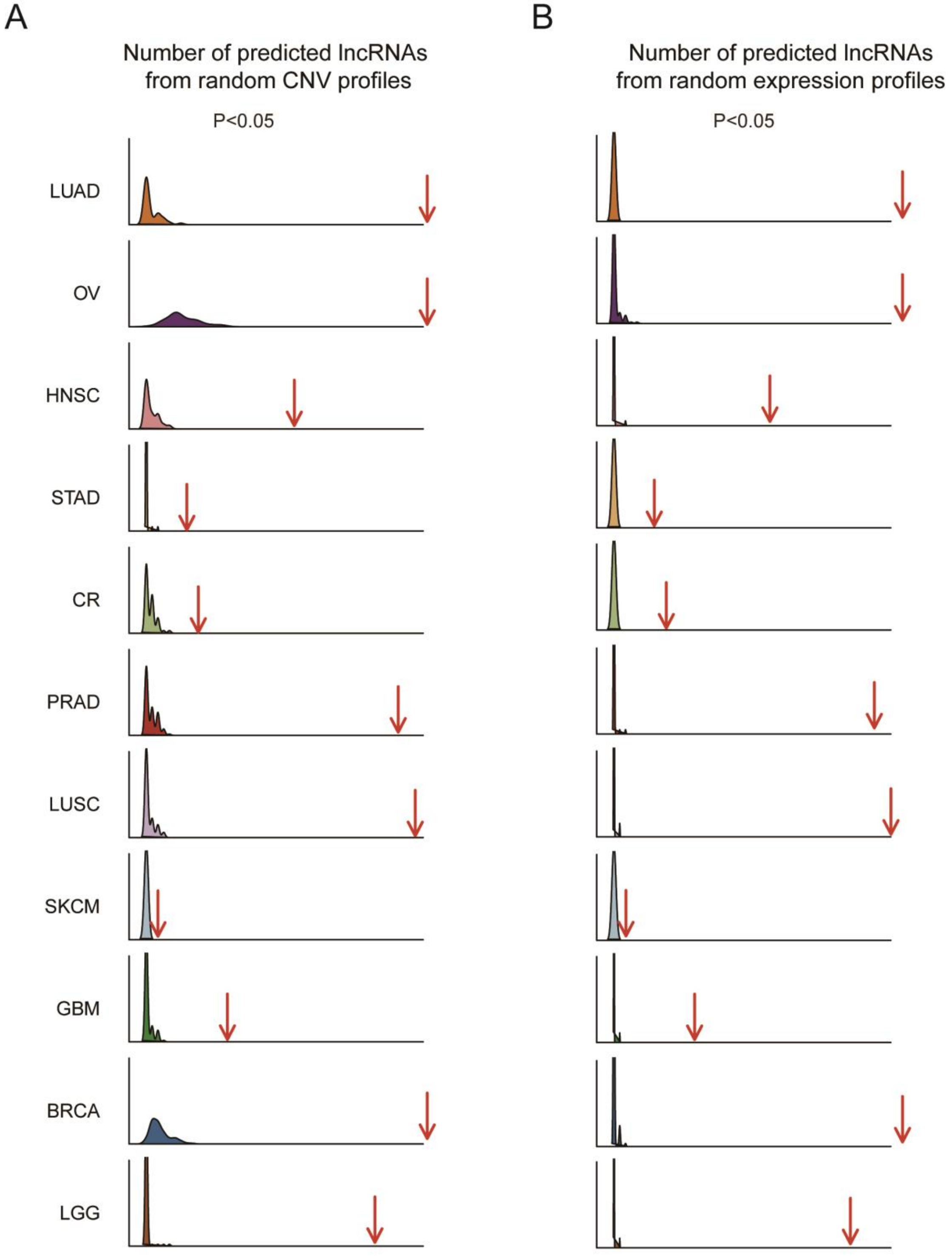
A
Number of predicted lncRNAs from random CNV profiles
P<0.05
LUAD
OV
HNSC
STAD
CR
PRAD
LUSC
SKCM
GBM
BRCA
LGG
B
Number of predicted lncRNAs from random expression profiles
P<0.05

Figure S7

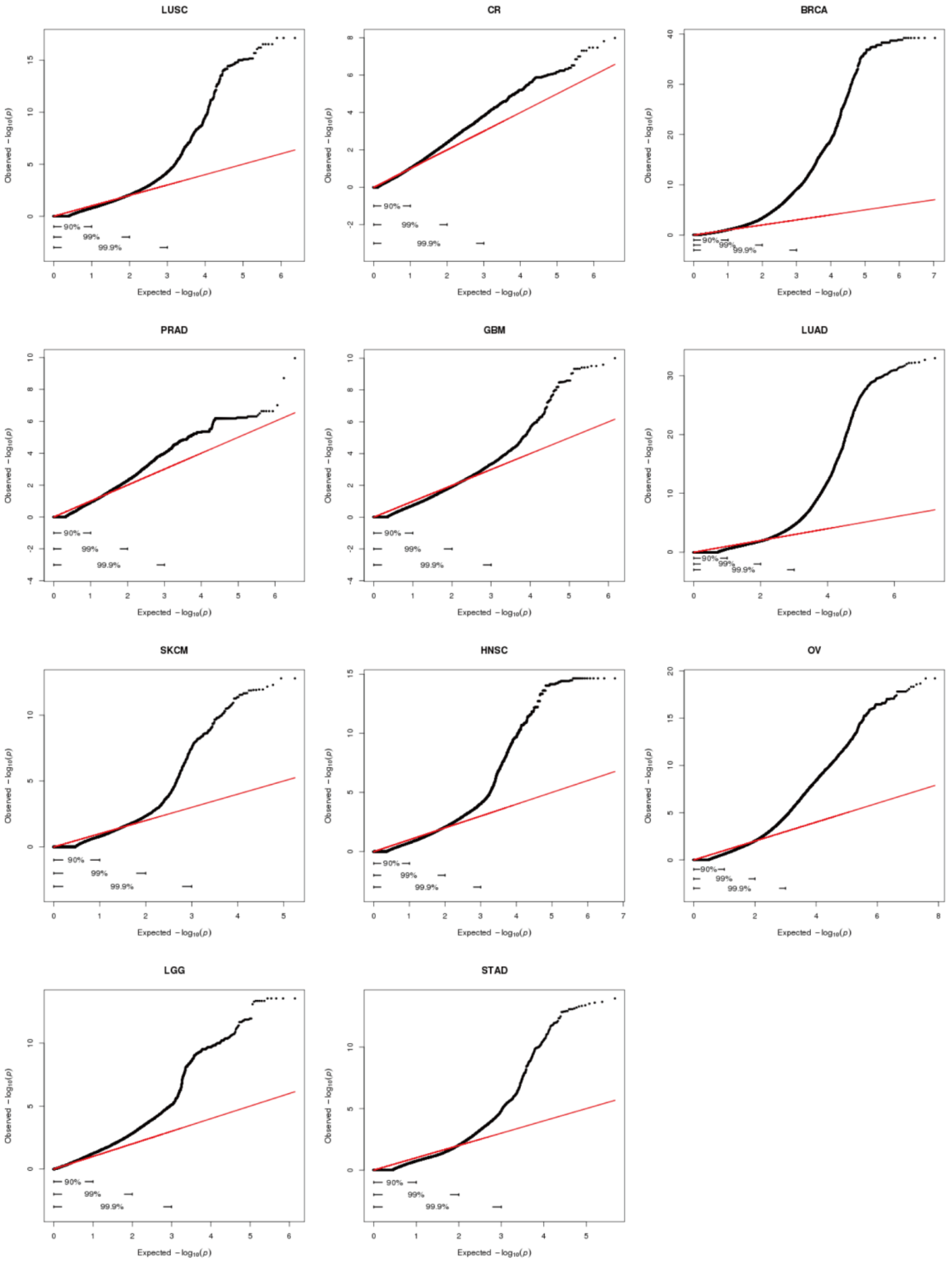
LUSC
CR
BRCA
PRAD
GBM
LUAD
SKCM
HNSC
OV
LGG
STAD
Observed $-\log_{10}(p)$
Expected $-\log_{10}(p)$
90%
99%
99.9%

Figure S8

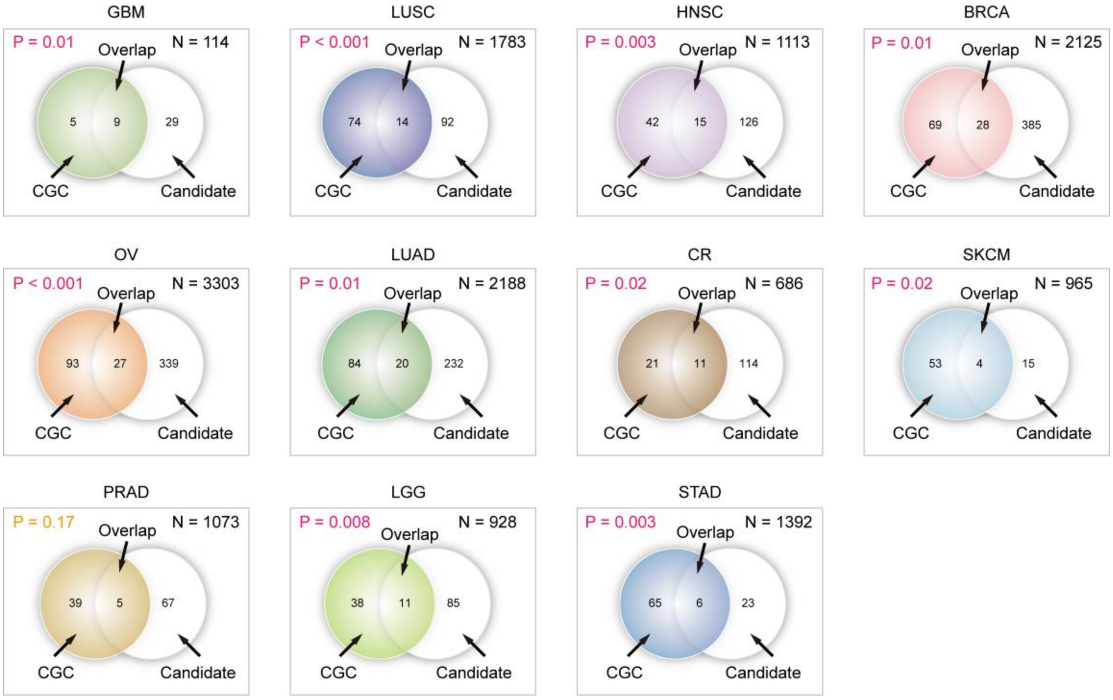

LUAD: lung adenocarcinoma HNSC: head and neck squamous cell carcinoma PRAD: prostate adenocarcinoma LGG: brain lower grade glioma STAD: stomach adenocarcinoma GBM: glioblastoma multiforme LUSC: lung squamous cell carcinoma BRCA: breast invasive carcinoma SKCM: skin cutaneous melanoma OV: ovarian serous cystadenocarcinoma CR: Colon adenocarcinoma&Rectum adenocarcinoma

Figure S9

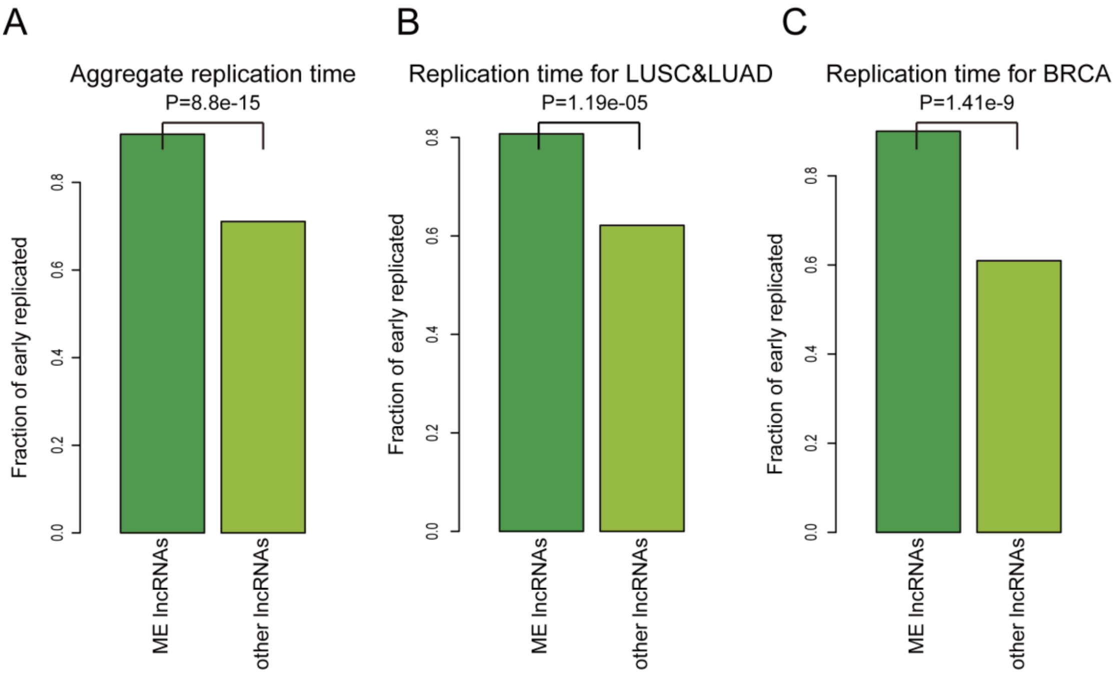

Figure S10

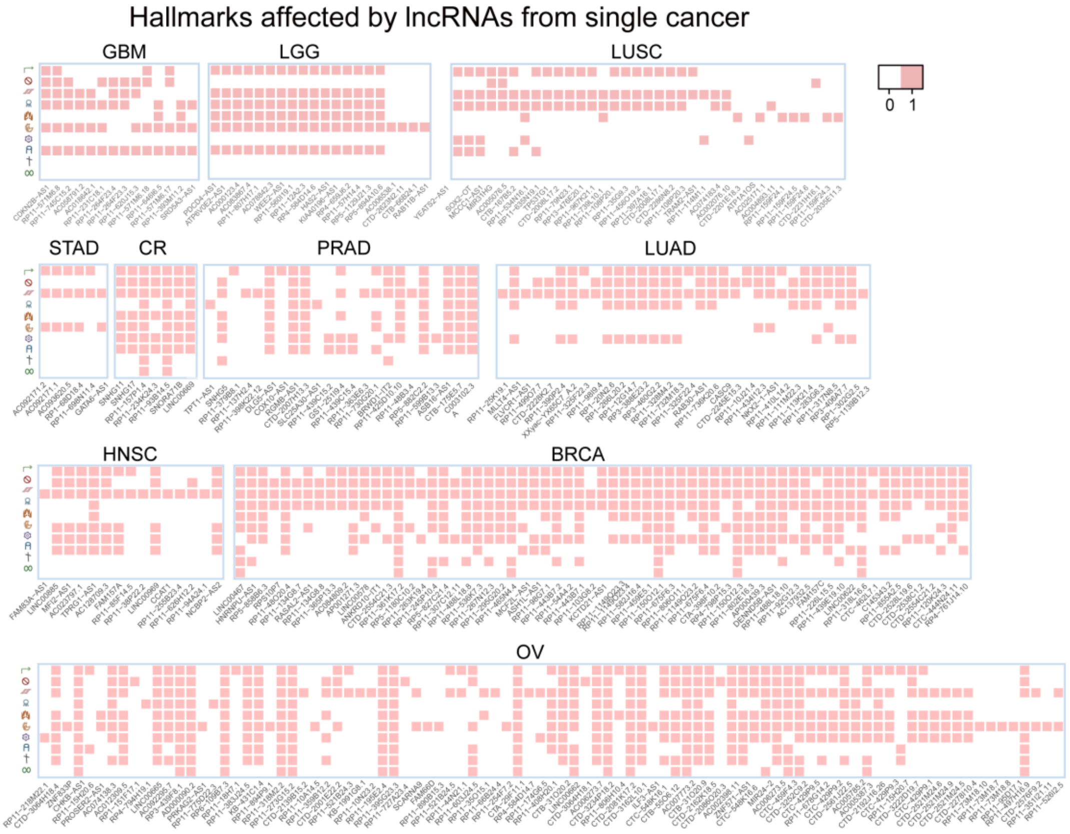
Hallmarks affected by lncRNAs from single cancer
GBM
LGG
LUSC
0
1
STAD
CR
PRAD
LUAD
HNSC
BRCA
OV

Figure S11

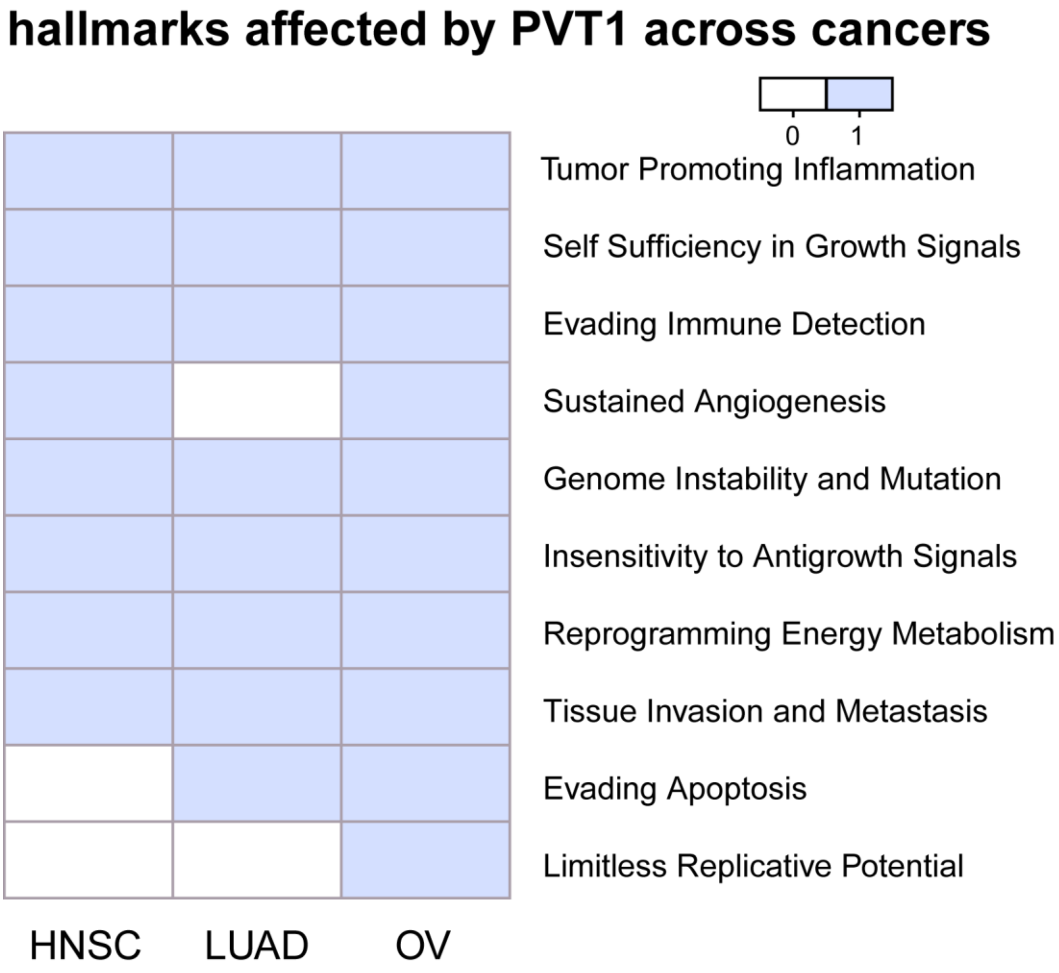

Figure S12

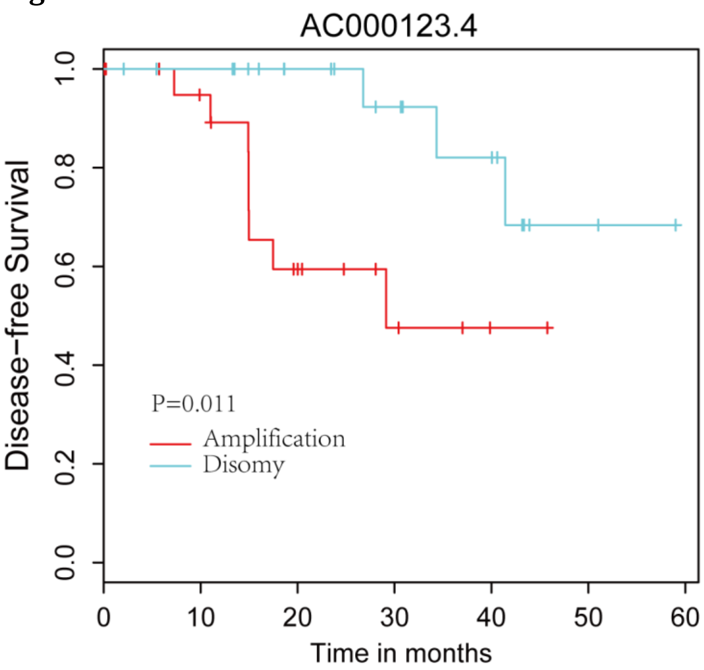

Supplement: Supplementary file 1 — Fig. S1. The distribution of semantic similarity score between candidate GO terms and hallmark‐associated GO terms. Fig. S2. Heatmaps show enrichment of hallmark‐associated PCGs in additional pathways that were downloaded from Synapse. Fig. S3. Construction of mutually exclusive networks from binary copy number alteration profiles. Fig. S4. The lncRNAs and protein‐coding genes in the top 10 most significant wide peak regions in BRCA, HNSC, LUAD, LUSC, PRAD, LGG, STAD, CR, SKCM and OV. Fig. S5. The basic statistics of mutually exclusive module across 11 major cancer types. Fig. S6. The number of predicted lncRNA from real data and random CNAs profiles and expression profiles across 11 cancer types. Fig. S7. QQ plot of P‐values that evaluate the hallmark associated function of mutually exclusive modules. Fig. S8. The overlap of candidate driver genes with Cancer Gene Census. Fig. S9. Proportion of lncRNAs from modules lie within early‐replicating regions. Fig. S10. Heatmaps show cancer‐associated hallmarks affected by cancer‐specific driver lncRNAs. Fig. S11. Heatmap shows cancer‐associated hallmarks affected by PVT1 in HNSC, LUAD and OV. Fig. S12. Kaplan–Meier plots of DFS of LGG patients grouped by copy number status of lncRNA AC000123.4. Table S1. Detailed information of patients analyzed in the present study. Table S2. The number of copy number altered PCGs and lncRNAs across cancers. Table S3. The number of mutually exclusive modules and driver lncRNAs across cancers. Table S4. The overlap of candidate driver lncRNAs identified by MiTranscriptome and our method. Table S5. The overlap of candidate driver lncRNAs identified by CRISPRi and our method. Table S6. Cox proportional hazards models of driver lncRNAs across cancers. Table S7. Univariate and multivariate Cox proportional hazards analysis of disease‐free survival for 429 brain lower grade glioma patients according to AC000123.4 copy number status. Table S8. Univariate and multivariate Cox proportional hazard [file MOL2-12-1980-s001.pdf]
